# The More Interest, the Less Effort Cost Perception and Effort Avoidance

**DOI:** 10.3389/fpsyg.2019.02146

**Published:** 2019-09-24

**Authors:** Juyeon Song, Sung-il Kim, Mimi Bong

**Affiliations:** ^1^Department of Education, Korea National University of Education, Cheongju, South Korea; ^2^Department of Education, Brain and Motivation Research Institute (bMRI), Korea University, Seoul, South Korea

**Keywords:** interest, effort cost, academic engagement, mathematics, gender difference

## Abstract

The present study aims to investigate what factors determine students’ engagement in mathematics. We examined the predictive relationships between interest, effort cost (i.e., the cost of making the effort), and three forms of academic engagement: persistence, cognitive engagement, and effort avoidance. In addition, we examined gender differences in these relationships. We recruited 546 8th and 9th graders for this study. Consistent with previous research, interest worked as a strong positive predictor of persistence and cognitive engagement, and it predicted effort avoidance negatively. Moreover, interest negatively predicted the perception of effort cost, which in turn positively predicted effort avoidance. Gender differences were found in the mean values of effort avoidance and in the prediction by interest of the perception of effort cost. Male students reported higher effort avoidance than female students, and the prediction by interest of the perception of effort cost was stronger among female students than among male students. These findings provide new insights into students’ engagement in mathematics and the role of interest and effort cost in it.

## Introduction

Many researchers and educators have been interested in how to make students study mathematics deeply and persistently. Interest is one of the most representative motivators that facilitate engagement (Eccles, 2016). Although interest researchers have slightly different definitions of interest, empirical evidence in a wide variety of theoretical frameworks consistently suggests that interest has a role in promoting academic engagement (Hidi et al., 2004; Sansone and Thoman, 2005; Reeve et al., 2015).

Recent motivation researchers have been trying to identify maladaptive motivators that hinder students’ academic engagement. Although previous research has identified trait procrastination and task difficulty as personality or environmental factors that hinder students’ engagement, there is still insufficient understanding of motivational factors that can explain negative motivation in academic engagement. One potential factor is effort cost which is defined as the perception of effort required to study mathematics (Battle and Wigfield, 2003; Jiang et al., 2018). Research studying the role of both positive and negative motivators together is expected to deepen our understanding of psychological mechanisms of students’ engagement in learning. Thus, the objective of this study was to examine the role of interest and effort cost together in predicting various forms of engagement.

Especially, gender differences in STEM (Science, Technology, Engineering, and Mathematics) areas have received worldwide attention. The previous studies have reported that boys are more likely than girls are to have positive motivation for math (Else-Quest et al., 2010). However, studies on gender differences in mathematics have focused primarily on the mean difference in math achievement and motivational variables, but few studies have examined whether male and female students have different or the same motivational paths in mathematics-related learning and decision-making. In addition, there is a report that the gender gap is decreasing even at the mean levels (Hyde and Linn, 2006; Gaspard et al., 2017). In this study, we thus aimed to examine whether gender differences still exist in the mean levels of math motivation and engagement and whether gender differences exist in the relationship between them.

## Theoretical Background

### Relationship Between Interest and Engagement

Academic engagement is defined as an investment into knowledge or skills that can yield meaningful in-depth learning (Newmann et al., 1992; Mayer, 2002). Students need to use cognitive strategies and persistent effort in order to produce meaningful learning. In this sense, persistent effort and cognitive strategy are viewed as the most representative forms of academic engagement (Greene and Miller, 1996; Fredricks et al., 2004). By contrast, effort avoidance is a different form of engagement, in which students participate in learning minimally with little meaningful learning taking place (Song et al., 2017). An example of this is an attitude that avoids trying to understand difficult parts or solve difficult problems.

The most representative role of interest is to increase deep levels of cognitive strategy uses and persistence. Interest is a motivational construct derived from inherent enjoyment that people feel in the process of performing a task (Schiefele, 1991; Sansone and Thoman, 2005). Interest has two distinct features. First, it can lead students to become intrinsically motivated and internally regulate their behavior (Ryan and Deci, 2000; Isen and Reeve, 2005). Second, it has a strong connection to positive emotion generated by engaging in a task (Higgins, 2006). The relationship between interest and engagement is reciprocal. Interest can be situationally triggered by engaging in a specific task. This type of the interest is called *situational interest* (Krapp, 1999; Hidi and Renninger, 2006). In this case, engagement seems to precede interest. The major role of situational interest is to focus attention. Situational interest may not last over time or in other situations (Hidi and Baird, 1986; Hidi, 1995). However, *individual interest*, which is defined as a relatively stable personal interest related to a particular domain, task, or activity, can function as high level of motivation and lead to the use of higher-order cognitive strategies with persistent engagement and learning (Krapp, 1999; Hidi and Renninger, 2006). In the present study, individual interest is postulated as a positive predictor for persistence or cognitive engagement based on Eccles’ Expectancy-Value Theory and Hidi and Renninger’s Interest Theory (Hidi and Renninger, 2006; Eccles, 2016).

Researches on the relationship between interest and effort avoidance are insufficient compared to studies on the relationship between interest and persistence or cognitive engagement. However, considering the positive role of interest in engagement, it can be expected that interest is negatively related to effort avoidance. In addition, investigating the relationships of effort cost with interest and effort avoidance will enable us to explain the negative link between interest and effort avoidance.

### Relationship Between Interest and Effort Cost

Effort consumes mental or physical energy. Thus, people tend to avoid participating in a task when it requires a large amount of efforts (Inzlicht et al., 2018). In this regard, effort is perceived as costly (Eccles et al., 1983). This negative perception of time, energy, or amount of work put into a task is named ‘effort cost’ (Eccles et al., 1983; Gaspard et al., 2015). In fact, effort and time are typically considered as primary costs when people make a decision (Botvinick et al., 2009; Vassena et al., 2014).

However, effort is not always perceived as costly. When the task is associated with feeling enjoyment, the effort may be no longer considered as costly (Inzlicht et al., 2018). This may due to two notable functions of interest: resource replenishment and effortless attention. First, Thoman et al. (2011) sought to explain why some people can engage in an interesting task even after their resources have been depleted; in a series of three studies, they discovered that interest has a resource replenishment function. In their research, participants were first depleted by a task (e.g., a Stroop task) and then asked to perform one of three emotionally stimulating tasks that evoked either interest, positive emotion, or neutral emotion. Following this, the participants then engaged in a subsequent, unrelated task for as long as they wanted to. Participants who had been given the interesting task persisted longer in the subsequent task than did those who had been asked to complete either the positive- or neutral-emotion task. Interestingly, this result was observed only when the participants’ energy had already been depleted before performing the second, emotionally stimulating task. The authors interpreted these differences in persistence as being a consequence of the resource-replenishment function of interest. They also tried to elucidate the underlying mechanism behind the resource-replenishment function and thus tested positive emotion and increased competence as potential mechanisms but were not able to identify the mechanism in question.

Automatic or effortless attention is another important function of interest. For example, a more-interesting text requires less time to read them than a less-interesting one, and people who read more-interesting texts perform better on recall tests than do those who read less-interesting texts (McDaniel et al., 2000). Individuals can quickly and effortlessly focus their attention on a target task when its characteristics, such as novelty and relevance, provoke their interest (Pekrun, 1992; McDaniel et al., 2000; Hidi et al., 2004; Hidi and Ainley, 2008). In this process, the cognitive effort or resources required to concentrate on a task can be preserved by automatic engagement and action. Effortless or automatic attention accordingly enables participants to focus more on deeper cognitive engagement (McDaniel et al., 2000; Linnenbrink and Pintrich, 2004).

Both resource replenishment and effortless attention are strongly connected to the effort or energy that individuals invest in a certain task. According to the research summarized above, interest allows for the recovery of previously drained energy levels through its resource-replenishment function and reduces cognitive effort itself by automatically drawing the attention of the participants. These studies provide an interesting insight into the relationship between interest and cost, especially effort cost. Considering the two functions of interest, it could be expected that interest would be a negative predictor of the perceived effort cost required to complete a task. In other words, even if people do the same amount of work, if they are interested in the task, they may be less aware of the effort required for the task. In addition, efforts combined with interest can be considered even valuable, rather than costly (Inzlicht et al., 2018). Despite this, few studies have examined the relationship between interest and effort cost. Some recent research has reported negative correlations between interest and effort cost, but the relationship between interest and effort cost was not a focus of the studies (Gaspard et al., 2015; Jiang et al., 2018).

Both interest and effort cost are students’ subjective perceptions rather than objective ones. Therefore, they can affect each other. For example, how much of a burden the effort required for a task feels like may depend on the degree of interest the individual has in the task, even if the task requires the same amount of effort. The opposite is also possible. One recent study has shown that task values, including interest, and costs can predict each other, although predictions differ depending on school years (Part et al., 2018), meaning that evidence for a causal relationship between interest and effort cost remains questionable. Therefore, in the present study, we first tried to explore whether there were students who had high interest and low effort costs, or if there were students who were both highly perceived by using a person-centered approach. Next, we sought to examine the role of interest in the perception of effort cost theoretically based on two functions of interest mentioned above. Specifically, interest in a task could lower the perception of effort cost because of the two functions of interest: resource replenishment and effortless attention (Hidi et al., 2004; Hidi and Ainley, 2008; Thoman et al., 2011).

### Relationship Between Effort Cost and Engagement

The perception of cost is found to be related to the intention to engage in a task and the intention to quit (Eccles and Wigfield, 1995; Battle and Wigfield, 2003; Kurzban et al., 2013; Perez et al., 2014). Avoidance-related intentions (e.g., intent to drop out) and behaviors (e.g., disengagement and procrastination) have been particularly identified as unique consequences of task costs such as effort cost (Perez et al., 2014; Jiang et al., 2018).

Although there were other costs such as opportunity cost and psychological cost, Flake et al. (2015) found that effort cost was the most frequent cost-related response (i.e., 42%) when students were asked to describe the features of the class which motivated them the least. Similarly, Perez et al. (2014) reported that only effort cost significantly and consistently predicted the intent to leave by STEM majors over time, whereas beliefs about competence, task value, opportunity cost, and psychological cost did not. Neuroscience research has also shown that individuals tend to avoid a highly demanding task when they can choose the task (Croxson et al., 2009; Kool et al., 2010; McGuire and Botvinick, 2010). Therefore, among task costs, the present study especially focused on effort cost for two reasons: (1) the unique functions of interest could be the rationale for the link between interest and effort cost, and (2) given the previous findings, effort cost seemed to show better prediction than other costs in the explanation of academic engagement. Linking the relationship between interest, effort cost, and engagement, interest is expected to directly and indirectly predict different forms of engagement by lowering the perception of effort cost.

### Gender Differences

According to a meta-study, there is no difference in math achievement between boys and girls, but boys are more likely to have high confidence, intrinsic motivation, and extrinsic motivation compared to girls (Else-Quest et al., 2010). Although there is no gender difference in the perception of values such as importance or usefulness, interest in mathematics has been somewhat consistently higher for male students than for female students (Pajares and Graham, 1999). However, recently, there has been a finding that there is no gender difference in interest (Gaspard et al., 2017). Therefore, there is still a need to accumulate more up-to-date data on gender differences in math motivation. Also, no gender difference in effort cost was found in the previous research.

Regarding gender difference in academic engagement, Hyde and Linn’s (2006) recent meta-study provided evidence for gender similarities in mathematics and science. They showed that there is no gender difference in mathematics grades and that there is no gender difference in complex problem solving in elementary and middle school. They say that in 2001, American women received 48% of the bachelor’s degrees in mathematics. These findings suggest that the motivation and persistence of men and women in mathematics may have reached similar levels. Furthermore, one study found that girls believed less about their math abilities than boys did, but displayed fewer work-avoidance goals, which aim to get as little involvement in a task as possible (Chouinard and Roy, 2008). This result means that boys’ engagement might be more likely to appear as maladaptive, such as effort avoidance, than does that of girls.

Gender differences mentioned above are about differences in mean levels. Like this, gender difference studies in mathematics have focused on differences in mean levels. Thus, there is less understanding of gender differences in the relationship between engagement and motivational beliefs such as interest and effort cost. Only some researchers have reported higher interest-achievement correlations in boys than in girls, meaning that boys are more influenced by their math interests than girls (Reeve and Hakel, 2000; Denissen et al., 2007). Therefore, it is meaningful to investigate whether there would be gender differences in the relationships between math interests, perceived effort cost, and engagement.

### Present Research

**FIGURE 1:**
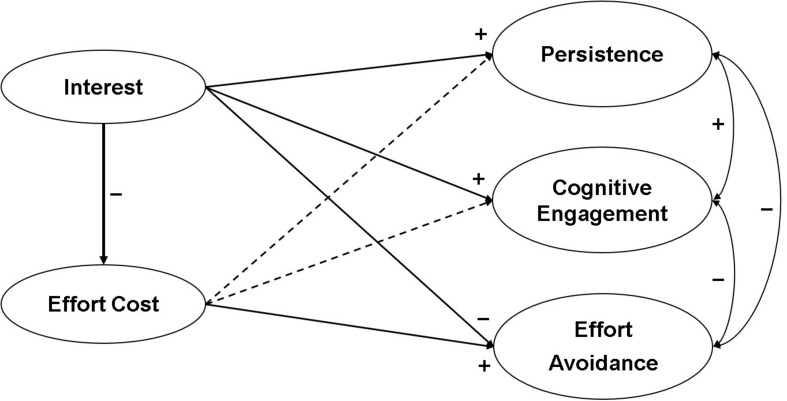
A hypothesized model. Dashed lines indicated a nonsignificant path. For clarity, indicators, measurement errors, and disturbance terms are not presented.

The purposes of this study were (1) to identify the role of interest and effort cost in persistence, cognitive engagement, and effort avoidance and (2) to examine gender differences in these roles. We preliminarily conducted a latent profile analysis to identify learners who had high interest and low perceived effort cost and who were high on both. We then used a structural equation model to test a hypothesized model. As Figure 1 shows, we hypothesized interest would positively predict persistence and cognitive engagement and negatively predict effort avoidance. By contrast, a recent research (Jiang et al., 2018) has shown that effort cost plays a more important role in predicting effort avoidance positively than in predicting persistence and cognitive engagement negatively. In addition, we tested a hypothesis that interest could negatively predict effort cost. Thus, effort cost was tested as a mediator especially in the relationship between interest and effort avoidance. Last, we run a multi-group analysis for the examination of gender differences in relationships among interest, effort cost, and engagement variables.

## Materials and Methods

### Participants

Five-hundred and sixty-three 8th and 9th graders from three middle schools in two metropolitan cities in South Korea voluntarily participated in this survey. The age of the students ranged from 13 to 16, and the average age was 14.12. Of the 563 students, six students did not complete the survey, and another eleven responded in a way that suggested their answers were insincere (e.g., choosing the same number for more than half the items in the entire survey). The size of the final sample used in this study was thus 546 (305 boys and 241 girls; 325 eighth graders and 221 ninth graders).

### Procedure

All the schools participated in the study were informed of the study and were given an opt-out option. The survey was administered during a regular class period. Parents were informed about the study through school announcement, and none of the parents raised doubts about this study. All participants voluntarily joined study and signed a written informed consent form. This survey study, collecting only students’ perceptions of learning and motivation without personal identifying information, did not include any vulnerable participants. The participants were also assured that their responses would not be disclosed to their parents or teachers but used only for research purposes. To reassure the students about the privacy of their responses, they sealed their questionnaire with a sticker after completing the survey. The research protocol was approved by the Institutional Review Board (IRB), Korea University.

### Measures

The students responded to all items using a 7-point Likert-type scale, where 1 indicated the strongest disagreement and 7 indicated the strongest agreement for all items. All items referred to mathematics, because the students were expected to have formed clear perceptions of the interest and effort cost associated with this subject (see Appendix). In addition, mathematics is a subject in Korea that is used to define academic success.

#### Interest

We used five interest items included in the Student Motivation in the Learning Environment Scale (SMILES; Bong et al., 2012) based on interest literature (Hidi and Harackiewicz, 2000; Hidi and Renninger, 2006). Reliability and validity of the scale have been systematically examined by using Korean sample in a previous research (Bong et al., 2012). In the validation study, the scale exhibited sufficient reliability (α = 0.94). Factor analysis supported its construct validity. High correlation of the SMILES interest scale with the existing interest scale (*r* = 0.77) supported its concurrent validity (Marsh et al., 2005). The reliability of the scale was acceptable in the present study (α = 0.85).

#### Effort Cost

For effort cost items were adopted from Gaspard et al. (2015), in which the scale reliability was reliable (ρ = 0.90). The scale produced a reliable Cronbach’s α coefficient (α = 0.87) in the present study.

#### Persistence

Persistence is measured with seven items (e.g., “If a mathematics problem is really hard, I keep working on it”), which were adopted from Miserandino (1996). The reliability was reported as 0.94 in the previous research using the original English version of the scale and reported as 0.89 in the previous research using the Korean version of the scale (Song et al., 2012). In the present study, the scale showed a reliable Cronbach’s α coefficient as 0.89.

#### Cognitive Engagement

Eight cognitive engagement items (e.g., “I try to understand rather than just memorize how to solve a mathematics problem”) were adopted from the cognitive engagement scale included in the SMILES (Bong et al., 2012). Cognitive engagement items were developed based on Bloom’s taxonomy and consisted of six levels of cognitive processing: remembering, understanding, applying, analyzing, evaluating, and creating (Krathwohl, 2002). Because the original scale items referred to academic study in general, all items in this study were thus revised to specifically refer to mathematics. The reliability of the scale was acceptable (α = 0.74).

#### Effort Avoidance

To measure effort avoidance, three items were adopted from Song et al. (2017) research. The validity and reliability of the scale has already been systematically tested (Song et al., 2017). The original items were revised to refer to mathematics. The scale produced a reliable Cronbach’s α coefficient (α = 0.80).

### Data Analysis

In this study, students were nested in 19 classes. Although multilevel analysis was not an original purpose of the present study, data had a complex structure. Thus, we applied design-based correction of standard errors to avoid underestimation of standard errors considering that the data had complex data structures (McNeish et al., 2017). The full information maximum likelihood (FIML) approach was applied to handle some missing data (0.0 to 0.88% of all items). We first conducted Latent Profile Analysis (LPA). The number of profiles was determined based on AIC (Akike Information Criterion), BIS (Bayesian Information Criterion), SABIC (Sample-size Adjusted BIC), adjusted LMR(Lo-Mendell-Rubin adjusted Likelihod Ratio Test), BLRT (Parametric Bootstrapped Likelihod Ratio Test), and entropy (McLachlan and Peel, 2000; Dias and Vermunt, 2006; Nylund et al., 2007).

Next, we conducted a structural equation modeling (SEM) with a whole sample in order to test the hypothesized model. Persistence and cognitive engagement scales have many items. They were parceled into three indicators after considering the small sample size compared to parameters to be estimated (Kline, 2011). Chi-square statistics, the Tucker-Lewis Index (TLI), comparative fit index (CFI), and the root mean square error of approximation (RMSEA) were used to evaluate the overall fit of the models. For the CFI and TLI, a coefficient above 0.90 indicates a suitable fit (Hu and Bentler, 1999) and for the RMSEA, values under 0.05 represent a close approximate fit, and values between 0.05 and 0.08 suggest an acceptable fit (Browne and Cudeck, 1993).

We then conducted a multi-group analysis to examine gender differences in predictive paths. For this, we tested measurement, covariance, and structural invariance over gender. To test measurement invariance, we constrained the factor loadings to be equal across gender. Then, we additionally constrained covariances between persistence, cognitive engagement, and effort avoidance to test covariance invariance. According to Chen (2007) and Cheung and Rensvold (2002), a decrease of less than 0.01 in the fit of the more parsimonious model on the CFI and TLI, an increase of less than 0.015 in the RMSEA, and an increase in the SRMR of 0.030 were considered as support for the more constrained model. Last, to test gender differences in path coefficients, we added constraints of the path coefficients one by one and tested the chi-square difference for every addition of a single path constraint. All analyses were conducted in Mplus 7.31.

## Results

### Descriptive Statistics and Correlations

Table 1 presents the descriptive statistics for all the scales. The presence of moderate mean scores, low skewness (less than |0.36|), and low kurtosis (less than | 0.53|) indicates that the scales all produced a range of scores that had an approximately normal distribution (Kline, 2011). The correlations between all latent variables are also presented in Table 1. As expected, interest was negatively correlated with effort cost (*r* = −0.42, *p* < 0.001). Interest was positively correlated with persistence (*r* = 0.81, *p* < 0.001) and cognitive engagement (*r* = 0.78, *p* < 0.001), and it was negatively correlated with effort avoidance (*r* = −0.47, *p* < 0.001). Conversely, effort cost was negatively correlated with persistence (*r* = −0.35, *p* < 0.001) and cognitive engagement (*r* = −0.27, *p* < 0.001), and it was positively correlated with effort avoidance (*r* = 0.51, *p* < 0.001). Persistence was highly and positively correlated with cognitive engagement (*r* = 0.97, *p* < 0.001). Effort avoidance was negatively correlated with persistence (*r* = −0.66, *p* < 0.001) and cognitive engagement (*r* = −0.53, *p* < 0.001).

**TABLE 1:** Descriptive statistics and latent correlations.

	**1**	**2**	**3**	**4**	**5**
1. Interest	–				
2. Effort cost	–0.42	–			
3. Persistence	0.81	–0.35	–		
4. Cognitive engagement	0.78	–0.27	0.97	–	
5. Effort avoidance	–0.47	0.51	–0.66	–0.53	–
*M*	3.73	3.86	4.41	4.34	3.31
*SD*	1.29	1.36	1.07	1.16	1.27
Skewness	0.04	0.08	–0.15	–0.30	0.36
Kurtosis	–0.42	–0.53	0.12	0.27	–0.12
α	0.85	0.87	0.89	0.74	0.80

**N* = 546. All correlation coefficients were significant at *p* < 0.001.*

### Latent Profiles

The number of profiles was determined based on AIC (Akike Information Criterion), BIS (Bayesian Information Criterion), SABIC (Sample-size Adjusted BIC), adjusted LMR(Lo-Mendell-Rubin adjusted Likelihod Ratio Test), BLRT (Parmametric Bootstrapped Likelihod Ratio Test), and entropy. As shown in Table 2, all indices supported a 4-profile solution. First, the lower the scores of AIC, BIS, and SABIC, the better the fit (Nylund et al., 2007). All three indices for each model are plotted in Supplementary Figure S1. AIC, BIC, and SABIC continued to go down as more latent profiles were added. However, slopes appeared to flatten out between 4 and 6 profiles. BLRT for each model was statistically significant. However, adjusted LMR was significant only up to four profiles, supporting the 4-profile solution (McLachlan and Peel, 2000). As seen in Supplementary Figure S2, entropy was also the highest at 0.849 in 4-profile solutions. Thus, the 4-profile classification was considered the most accurate (Dias and Vermunt, 2006).

**TABLE 2:** Latent profile solutions.

***N* of Profile**	**AIC**	**BIC**	**Adjusted BIC**	**Adjusted LMR**	**Entropy**	**Class sizes (%)**					
2	7097.035	7165.877	7115.087	<0.001	0.775	0.53	0.47				
3	6773.654	6868.312	6798.475	0.141	0.845	0.16	0.60	0.24			
**4**	**6591.932**	**6712.406**	**6623.523**	**0.052**	**0.849**	**0.13**	**0.39**	**0.10**	**0.37**		
5	6554.862	6701.151	6593.222	0.485	0.821	0.10	0.16	0.32	0.37	0.05	
6	6512.120	6684.225	6557.249	0.331	0.837	0.03	0.09	0.15	0.35	0.05	0.33

*The final selected model is shown in bold.*

**FIGURE 2:**
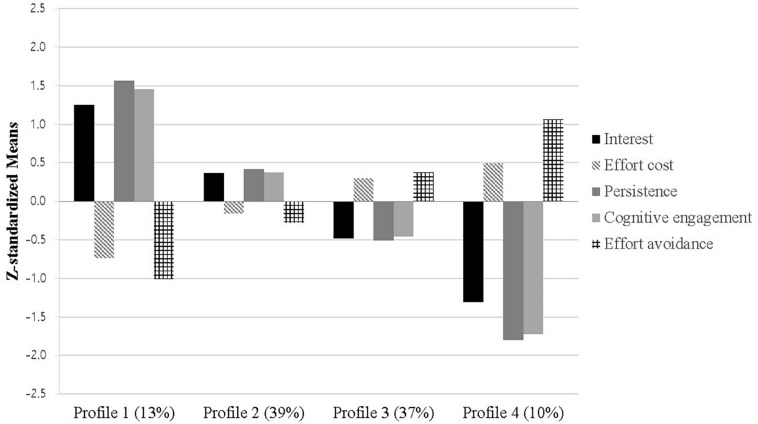
Z-standardized means of profiles.

As expected, interest, persistence, and cognitive engagement were either all high or all low. Mean levels of effort cost and effort avoidance appeared to be opposite to them. As shown in Figure 2, four profiles were identified. Profile 1 showed high levels of interest, persistence, and cognitive engagement but low levels of effort cost and effort avoidance. Students in this profile 1 seemed to have the most positive perceptions of math. Both Profile 2 and 3 displayed moderate levels in all variables. Profile 2 displayed relatively higher levels of interest, persistence, and cognitive engagement but lower levels of effort cost and effort avoidance than the average, whereas Profile 3 presented relatively lower levels of interest, persistence, and cognitive engagement but higher levels of effort cost and effort avoidance than the average. Lastly, Profile 4 showed low levels of interest, persistence, and cognitive engagement but high levels of effort cost and effort avoidance.

### Test of the Hypothesized Model

The model showed acceptable fit to the data, χ^2^ (125, *N* = 546) = 376.576, *p* < 0.001 (CFI = 0.938, TLI = 0.924, RMSEA = 0.061, SRMR = 0.056). As Table 3 shows, consistent with the hypotheses, interest positively predicted persistence (β = 0.80, *p* < 0.001) and cognitive engagement (β = 0.82, *p* < 0.001) and negatively predicted effort avoidance (β = −0.31, *p* < 0.001). Moreover, interest negatively predicted effort cost (β = −0.42, *p* < 0.001). Effort cost did not predict either persistence (β = −0.02, *p* = 0.584) or cognitive engagement (β = 0.08, *p* = 0.153), but positively predicted effort avoidance (β = 0.38, *p* < 0.001). By doing so, effort cost significantly mediated the path from interest to effort avoidance (β = −0.16, *p* < 0.001, see Table 4). The standardized path coefficients of the total effect of interest on effort cost was thus −0.47 (*p* < 0.001). Persistence and cognitive engagement were highly correlated with each other (*r* = 0.93, *p* < 0.001). For robustness check, we examined whether similar results were found when excluding either persistence or cognitive engagement from the hypothetical model. As a result, model fits were similar. Significance of path coefficients did not change.

**TABLE 3:** Results from SEM and multi-group analysis.

	**Whole**			**Female students**			**Male students**		
**Path**	**β**	***SE***	***p***	**β**	***SE***	***p***	**β**	***SE***	***p***
Interest → Effort cost	–0.422	0.06	<0.001	–0.663	0.05	<0.001	–0.278	0.07	<0.001
Interest → Persistence	0.802	0.04	<0.001	0.784	0.05	<0.001	0.805	0.03	<0.001
Effort cost → Persistence	–0.016	0.03	0.584	–0.024	0.03	0.479	–0.023	0.03	0.480
Interest → Cog eng	0.815	0.04	<0.001	0.786	0.06	<0.001	0.843	0.04	<0.001
Effort cost → Cog eng	0.075	0.05	0.153	0.081	0.06	0.160	0.081	0.06	0.160
Interest → Eff avoid	–0.307	0.05	<0.001	–0.297	0.05	<0.001	–0.299	0.05	<0.001
Effort cost → Eff avoid	0.380	0.05	<0.001	0.390	0.05	<0.001	0.367	0.05	<0.001
Persistence ↔ Cog eng	0.928	0.03	<0.001	0.901	0.05	<0.001	0.956	0.05	<0.001
Persistence ↔ Eff avoid	–0.572	0.07	<0.001	–0.706	0.08	<0.001	–0.541	0.05	<0.001
Cog eng ↔ Eff avoid	–0.374	0.06	<0.001	–0.414	0.055	<0.001	–0.387	0.06	<0.001
***R*^2^**									
Effort cost	0.178	0.05	<0.001	0.439	0.06	<0.001	0.077	0.04	0.038
Persistence	0.655	0.06	<0.001	0.639	0.07	<0.001	0.658	0.05	<0.001
Cog eng	0.618	0.05	<0.001	0.540	0.06	<0.001	0.679	0.05	<0.001
Eff avoid	0.337	0.04	<0.001	0.394	0.06	<0.001	0.285	0.04	<0.001

*Cog eng, cognitive engagement; Eff avoid, effort avoidance.*

**TABLE 4:** Total, direct, and indirect effects of indirect paths.

			**Total**			**Direct**			**Indirect**		
**Path**			**β**	***SE***	***p***	**β**	***SE***	***p***	**β**	***SE***	***p***
Interest → Effort cost	→	Persistence	0.81	0.03	<0.001	0.80	0.04	<0.001	0.01	0.01	0.577
	→	Cog eng	0.78	0.03	<0.001	0.82	0.04	<0.001	–0.03	0.02	0.169
	→	Eff avoid	–0.47	0.05	<0.001	–0.31	0.05	<0.001	–0.16	0.03	<0.001
***Female***											
Interest → Effort cost	→	Persistence	0.81	0.03	<0.001	0.81	0.03	<0.001	0.01	0.01	0.439
	→	Cog eng	0.82	0.03	<0.001	0.84	0.04	<0.001	–0.02	0.02	0.240
	→	Eff avoid	–0.40	0.05	<0.001	–0.30	0.05	<0.001	–0.10	0.03	<0.001
***Male***											
Interest → Effort cost	→	Persistence	0.80	0.05	<0.001	0.78	0.05	<0.001	0.02	0.02	0.479
	→	Cog eng	0.73	0.04	<0.001	0.79	0.06	<0.001	–0.05	0.04	0.164
	→	Eff avoid	–0.56	0.05	<0.001	–0.30	0.05	<0.001	–0.26	0.04	<0.001

*Cog eng, cognitive engagement; Eff avoid, effort avoidance.*

### Tests of Gender Differences

Table 5 shows descriptive statistics for each female and male sample. The presence of moderate mean scores, low skewness, and low kurtosis support that the scales all produced a range of scores that had an approximately normal distribution in both the female and the male sample (Kline, 2011). Gender difference was found only in the mean value of effort avoidance (*t* = 3.04, *p* = 0.003).

**TABLE 5:** Descriptive statistics and mean difference by gender.

	**Female students (*n* = 241)**					**Male students (*n* = 305)**						
**Variable**	***M***	***SD***	***S***	***K***	**α**	***M***	***SD***	***S***	***K***	**α**	***t***	***d***
Interest	3.63	1.19	0.11	−0.16	0.85	3.80	1.35	−0.03	−0.58	0.87	1.54	0.13
Effort cost	3.79	1.28	0.03	−0.30	0.87	3.93	1.37	0.08	−0.60	0.87	1.22	0.11
Persistence	4.37	1.04	−0.02	−0.06	0.90	4.44	1.09	−0.24	0.25	0.89	0.76	0.07
Cognitive engagement	4.38	0.93	0.03	0.17	0.83	4.38	1.00	−0.27	0.39	0.86	0.12	0.00
Effort avoidance	3.12	1.21	0.27	−0.58	0.80	3.45	1.30	0.40	0.06	0.79	3.04^∗^	0.26

*S, skewness; K, kurtosis. ^∗^*p* < 0.01.*

Multi-group analysis was performed to investigate gender differences in the predictive relationships in the model. First, we checked measurement invariance by constraining all factor loading to be equal. As shown in Table 6, the measurement invariance model (Model 1) fitted the current data well, χ^2^(281, *N* = 546) = 759.25, *p* < 0.001 (CFI = 0.920, TLI = 0.913, RMSEA = 0.079, SRMR = 0.068). We next tested a covariance invariance model by constraining covariance invariances between persistence, cognitive engagement, and effort avoidance to be equal (Model 2). Compared to the measurement invariance model, no decreases in the GFI and TLI and the low increases in the RMSEA and SRMR (ΔCFI = 0.000, ΔTLI = 0.001, ΔRMSEA = 0.000, ΔSRMR = 0.001) provide support for the covariance invariance model, indicating there were no gender differences in the covariance coefficients.

**TABLE 6:** Model fit statistics for a multi-group analysis.

**M**	**Hypothesis**	**χ^2^**	***df***	**CFI**	**TLI**	**RMSEA**	**SRMR**	**Δχ^2^**
1	Measurement invariance	637.104	281	0.921	0.914	0.068	0.068	
2	Covariance invariance	638.856	284	0.921	0.915	0.068	0.069	
3	Interest → Effort cost	657.751	285	0.917	0.911	0.069	0.082	18.90^∗∗^
4	Interest → Persistence	658.403	286	0.917	0.912	0.069	0.082	0.65
5	Effort cost → Persistence	661.924	287	0.917	0.911	0.069	0.088	3.52
6	Interest → Cog eng	663.975	288	0.917	0.911	0.069	0.090	2.05
7	Effort cost → Cog eng	665.721	289	0.916	0.912	0.069	0.091	1.75
8	Interest → Eff avoid	666.789	290	0.916	0.912	0.069	0.093	1.07
9	Effort cost → Eff avoid	668.118	291	0.916	0.912	0.069	0.092	1.33
10	Final model	646.462	290	0.921	0.917	0.067	0.071	

*M, model; Cog eng, cognitive engagement; Eff avoid, effort avoidance. ^∗∗^*p* < 0.001.*

**FIGURE 3:**
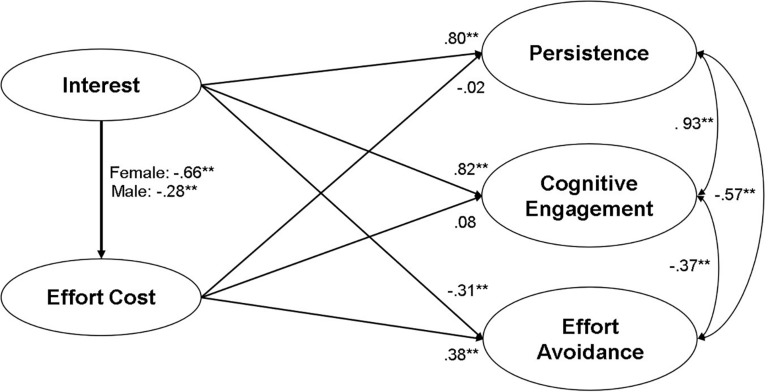
Results from SEM and multi-group analysis. The standardized coefficients for male and female students are separately presented only for the interest-effort cost path that is significantly different between the two. For clarity, indicators, measurement errors, and disturbance terms are not presented. ^∗∗^*p* < 0.001.

To examine gender difference in regression coefficients, we consecutively compared the chi-square of models from Model 3 to Model 9. For example, to test gender difference in the path from interest to effort cost, we additionally constrained the path to be equal across gender (Model 3) and compared the chi-square of Model 3 with that of Model 2, in which all paths were freely estimated but factor loadings and covariance were equally constrained (Δ*df* = 1). To test gender difference in the path from interest to persistence, we again additionally constrained the path to be equal (Model 4) and compared the chi-squares between Model 3 and Model 4 (Δ*df* = 1). For all seven path coefficients, gender differences were examined in the same way, and gender differences were found only in the path from interest to effort cost. Table 3 presents results from the final model. As Figure 3 shows, the prediction of interest for effort cost was much larger for female students (β = −0.66, *p* < 0.001) than for male students (β = −0.28, *p* < 0.001).

## Discussion

The present study examined the predictive relationship between interest, effort cost, and academic engagement. We also verified if there are gender difference. Frist, consistent with previous interest research (Schiefele, 1992; Mayer, 1998; Renninger et al., 2002), we observed that interest played a significant role in predicting students’ persistence and cognitive engagement. We further found that interest negatively predicted effort avoidance as well. Second, interest negatively predicted effort cost, which in turn positively predicted effort avoidance, indicating that effort cost mediated the relationship between interest and effort avoidance. In addition, a latent profile analysis supported our hypothesis. Levels of interest and effort costs varied depending on the type of profiles. If one was high, the other was low. Lastly, we found that boys were more likely to avoid effort than were girls. Apart from this, there was no difference between boys and girls in the mean of interest, effort cost, persistence, and cognitive engagement. Rather, the negative prediction for the perception of effort cost by interest was found to be even greater for girls than for boys.

### Importance of Interest in Academic Engagement

Interest has been identified as a potent motivator, containing both emotional and cognitive aspects. Individual interest for mathematics is developed by both enjoyment and internalizing importance of the task, which is positively linked to various forms of student engagement, such as persistence, effort, deep-levels of cognitive engagement, and classroom engagement (Sansone and Thoman, 2005; Hidi and Renninger, 2006; Kim et al., 2015). In particular, cognitive engagement using a wide range of cognitive strategies from memorizing to critical thinking is essential to fully understand the learning material (Krathwohl, 2002; Mayer, 2002). Our study showed that interest was largely related to cognitive engagement, covering a full spectrum of cognitive processing such as outlining knowledge, applying knowledge to real life, and connecting new and old knowledge, consistent with previous studies (Walker et al., 2006; Wang et al., 2017). Our finding that interest positively predicts persistence also demonstrates that math interest of students helps them continue their studies even if they have difficulty in studying mathematics. Furthermore, interest is negatively linked to effort avoidance, which is a maladaptive form of engagement (e.g., minimizing effort investment and skipping difficult parts). That is, if students are less interested in mathematics, they may be reluctant to put more effort into math learning.

In sum, math interest plays a pivotal role in studying mathematics because it plays two important roles together: positively predicting persistence and cognitive engagement and negatively predicting effort avoidance. Given the strong connection between interest and engagement, researchers and educators need to help students to develop their interest in mathematics by making the learning environment more enjoyable.

### Effort Cost as an Avoidance Motivation

Among various forms of cost (e.g., effort cost, opportunity cost, and psychological cost) effort cost has been identified as the most salient feature of their least motivating classes. Example statement was “*these courses are too intense, requiring too much time, or being too rigorous*” (Flake et al., 2015). Perez et al. (2014) also discovered that only effort cost was strongly involved in the intent to leave a STEM major, whereas task value did not contribute to this decision.

Effort cost seems to be more deeply involved in avoidance motivation. Previous research has also observed that effort cost serves as a motivation to avoid the least motivating classes or to leave courses and a STEM major (Eccles et al., 1983; Croxson et al., 2009; Perez et al., 2014; Flake et al., 2015). Consistent with previous findings, effort cost positively predicted effort avoidance only. Findings from this study thus support previous findings that a task-related cost is a precursor of avoidance motivation and has been related to avoidance-oriented maladaptive behaviors and negative emotions, such as test anxiety, negative classroom affect, disorganization, and procrastination (Jiang et al., 2018).

### Negative Relationship Between Interest and Effort Cost

When considering the detrimental role of effort cost in motivation, the negative relationship between interest and effort cost is particularly noteworthy. This inverse relationship was supported by both person-centered and variable-centered approaches. We found four different profiles based on five variables (interest, effort cost, persistence, cognitive engagement, and effort avoidance). In all four profiles, students did not have similar levels of interest and effort cost. Rather, levels of interest and effort cost were opposite.

Moreover, as expected, interest appeared as a negative predictor of effort cost perception, although this finding was based on cross-sectional data. It can be conjectured that replenishing energy, automatic attention, or both may be potential mechanisms of interest in predicting perceptions of effort cost. However, the present study did not directly examine this. The negative relationship between interest and effort cost might be due to an unknown third variable. For example, task difficulty can increase effort cost while reducing interest (Eccles et al., 1983; Inzlicht et al., 2018). Therefore, further research is needed to explain the negative relationship between interest and effort costs. Nevertheless, this study is still significant because it reveals a direct connection between interest and effort cost.

### Gender Differences

Interestingly, the negative relationship between interest and effort cost is more evident in female students. In previous studies, there was a higher correlation between interest and achievement or knowledge for males than for females (Reeve and Hakel, 2000; Denissen et al., 2007). Denissen et al. (2007) assumed that the low correlation between interest and achievement arises because female students focus on all areas to achieve overall high achievement rather than on the one area in which they are most interested and building a deeper level of knowledge of it. However, given the possibility that interest lowers perception of effort cost, female students’ math interest also plays a meaningful role in mathematics-related learning and decision making. Especially, many researchers are trying to find out why female students are leaving the STEM area, including mathematics, and effort cost is supposed to be one of the factors that predict intent to leave (Eccles et al., 1983; Perez et al., 2014). The findings suggest that it is necessary to consider both positive values (e.g., interest) and negative costs (e.g., effort cost) to understand female students’ engagement in mathematics.

When it comes to mean differences, there was no gender difference in the mean levels of all variables except for effort avoidance. Previous research has shown that there is no difference between male and female students in actual achievement, but there are gender differences in motivational beliefs in mathematics, such as interest and beliefs about competence (Else-Quest et al., 2010). Gender stereotypes have been identified as one core factor among the possible causes of these gender differences in mathematics (Jacobs, 1991; Spencer et al., 1999). However, it is not only our research that has not found gender differences in math beliefs. Recent German data has also reported that there is no gender difference in expectancy and value beliefs in mathematics (Gaspard et al., 2017). More research is thus needed to confirm whether gender stereotypes are socially weakened and gender differences are decreasing in students’ subjective beliefs about mathematics as well as in their actual achievement.

This study even showed that boys are more likely than girls are to show maladaptive patterns of engagement, such as skipping hard part. This is in line with the existing literature that showed boys are more likely to pursue work-avoidance goals in mathematics than girls are (Chouinard and Roy, 2008). It is therefore necessary to accumulate up-to-date data on recent changes in beliefs about mathematics, apart from past findings of gender differences. Nevertheless, there are still some recent studies reporting gender differences in mathematics (Tian et al., 2018). Considering this, research will need to be conducted to examine gender differences in various samples and contexts as to which elements reduce gender differences.

### Limitations and Future Directions

Considering the limitations of this study, we suggest future research directions as follows. First, based on the theoretical background, we hypothesized that interest leads to the perception of effort cost, but we measured all variables at one point. In fact, there is little research that draws conclusions about the temporal relationship or causal relationship between interest and effort cost, so further experimental and longitudinal studies should be accumulated. Also, effort cost has emerged as an important factor in academic motivation and choice-related behaviors, especially in avoidance-related behaviors such as procrastination (Perez et al., 2014; Jiang et al., 2018). In addition to interest, more systematic research is needed on what factors lower the perception of effort cost. Basically, it will be influenced by the absolute amount of the task, but it will also be affected by actual characteristics of the task, such as task difficulty, and other subjective perceptions about the task, such as belief in one’s competence (Eccles et al., 1983). It is also necessary to further examine whether other types of task value, such as utility value, play the same role in effort cost perception as did interest.

## Conclusion

The current study has several theoretical and educational implications. It also presents a new perspective on the role of interest in relation to effort cost. First, the present research makes a contribution to interest literature by demonstrating a new role of interest. Although previous studies have shown that interest plays a significant role in promoting academic engagement (Schiefele, 1992; Durik et al., 2006; Walker et al., 2006), our findings suggest that interest is an important predictor of both effort cost perception and effort avoidance. Second, the current study shows that there is a need to pay more attention to the role of effort cost. The classic expectancy-value theory has focused only on task values such as interest and utility value, but not on task costs (e.g., Eccles et al., 1983; Wigfield and Eccles, 2000). Although traditional studies have clearly shown that task values function as a critical factor in predicting achievement-related outcomes, there is little research on cost perception. Recently, researchers have begun to propose the new Expectancy-Value-Cost (EVC) approach which highlights the importance of cost perceptions in learning process (Jiang et al., 2018). The present study further showed that effort cost played a mediating role between interest and effort avoidance, providing additional empirical evidence for supporting the recent EVC approach.

This study also has practical implications for educators. Given the strong relationship between interest, effort cost, and engagement, teachers and parents need to be aware of the importance of interest. Especially, considering the stronger link between interest and effort cost for female students, more attention should be paid to their interest in mathematics. Since girl’s math interest can be affected by parents and teachers’ gender-related math attitudes and stereotypes through their behaviors and communications (Gunderson et al., 2012), teachers and parents need to be careful about their words and actions so that they would not negatively affect girls’ interest in mathematics.

## Ethics Statement

All study participants provided informed consent, and the research protocol was approved by the Institutional Review Board (IRB), Korea University.

## Author Contributions

JS, SK, and MB designed the research and have contributed to writing the manuscript. JS collected and analyzed the data.

## Conflict of Interest

The authors declare that the research was conducted in the absence of any commercial or financial relationships that could be construed as a potential conflict of interest.

## Appendix

### Interest

I find mathematics interesting.

I lose track of time when I study mathematics.

I want to learn more about mathematics outside of class.

I feel happy when I learn new things related to mathematics.

I want to have a mathematics-related career.

### Effort Cost

Dealing with mathematics drains a lot of my energy.

Doing math is exhausting to me.

I often feel completely drained after studying mathematics.

Learning mathematics exhausts me.

### Persistence

If a mathematics problem is really hard, I keep working at it.

If I can’t understand mathematics contents right the first time, I just keep trying.

If I can’t think of the answer to a mathematic question, after minutes it comes to me.

I really concentrate when my teacher presents new materials in mathematics.

When I have trouble with a mathematics problem, I usually get it right in the end.

When I get stuck on a mathematics question, I can usually get it.

I pay attention when I start a new subject in mathematics.

### Cognitive Engagement

When I study mathematics, I explore out alternative solutions.

I question the validity of what I have learned in mathematic.

When I study mathematics, I distinguish main points from details.

I outline the learning materials that I learned in mathematics.

I apply what I learned in class while I do exercises.

I try to understand rather than just memorize the way to solve a mathematics problem.

When I learn something new in mathematics, I try to connect it to what I already know.

I try to remember what I have learned in mathematics as many as possible.

### Effort Avoidance

I solve only easy problems while I do exercises.

When studying mathematics, I skip all the hard parts.

If I cannot understand the learning materials in mathematics, I just skip it.
