# Supplementary material for: The More Interest, the Less Effort Cost Perception and Effort Avoidance
Source: Front Psychol. 2019 Sep 24;10:2146. doi: 10.3389/fpsyg.2019.02146 (PMC6769126; doi:10.3389/fpsyg.2019.02146)
Supplement: Supplementary file 1 [file Image_1.pdf]

Supplementary Material

1 Latent Profile Analysis

We conducted a latent profile analysis to find out what kind of learners were identified and how the relationship between the variables appeared within the individual.

1.1 Supplementary Figures

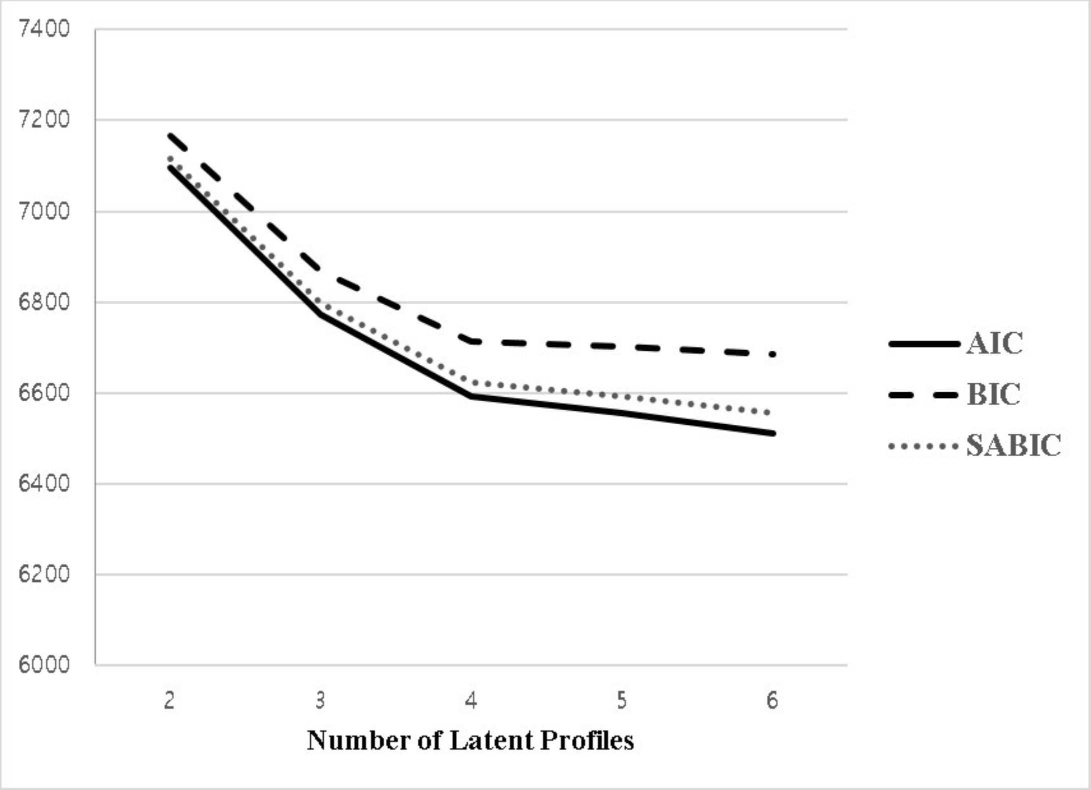

Supplementary Figure 1. AIC, BIC, and SABIC for each model. The lower the scores in the AIC, BIS, and SABIC, the better the fit (Nylund, Asparouhov, & Muthén, 2007). The AIC, BIC, and SABIC continue to go down as more latent profiles are added, but the slopes appear to flatten out between 4 and 7 profiles.

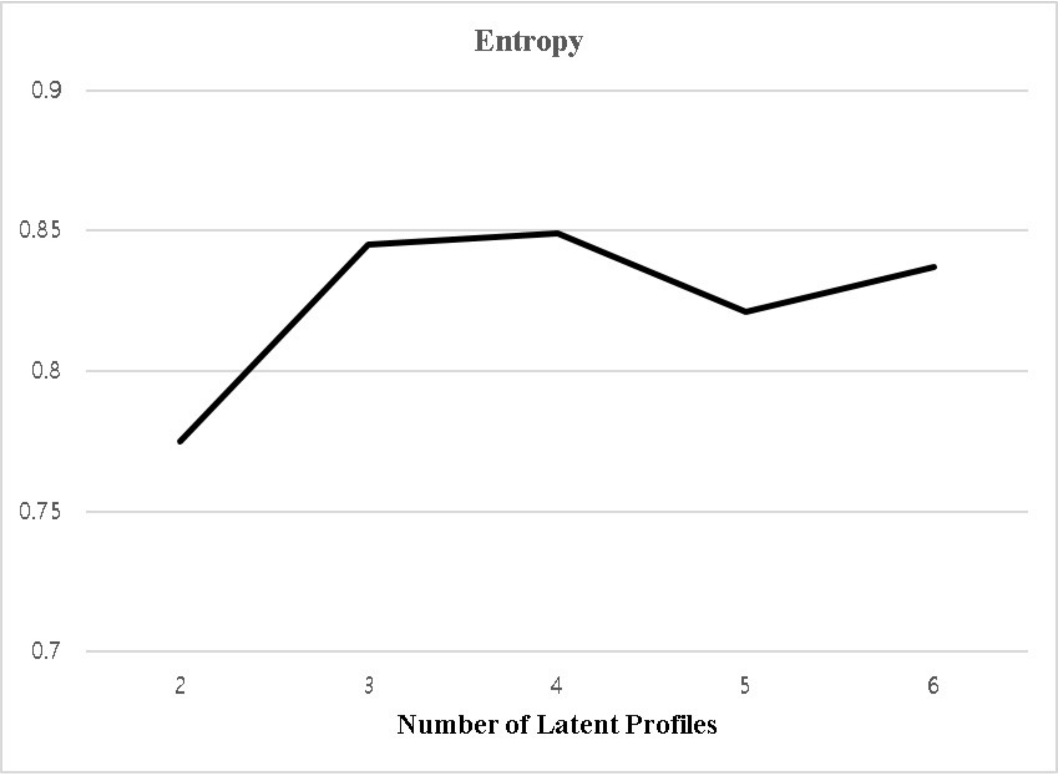

Supplementary Figure 2. Entropy for each model. Entropy was also the highest as .849 in 4-profile solutions, so the 4-profile classification can be considered the most accurate (Dias & Vermunt, 2006).

2 Covariance Matrices

Each covariance matrix for all, female, and male samples was given in order below. The variable names used in the analysis are as follows: II = interest; EFC = effort cost; PRS = persistence, CENG = cognitive engagement; EFFV = effort avoidance.

2.1 Covariance Matrix for the Whole Sample

	II01	II02	II03	II04	II05
II01	2.955				
II02	1.828	2.795			
II03	1.401	1.306	2.252		
II04	1.612	1.456	1.400	2.865	
II05	1.420	1.369	1.139	1.044	2.455
EFC01	-0.499	-0.319	-0.186	-0.231	-0.140
EFC02	-1.259	-1.099	-0.778	-0.999	-0.765
EFC03	-0.809	-0.632	-0.312	-0.320	-0.408
EFC04	-1.044	-0.831	-0.509	-0.522	-0.594
PRS01	1.348	1.238	0.882	1.019	0.996
PRS02	1.109	0.948	0.759	0.753	0.842
PRS03	1.124	0.911	0.792	0.810	0.752
PRS04	1.144	1.165	0.980	0.931	1.101
PRS05	1.180	0.930	0.827	0.904	0.759
PRS06	1.017	0.842	0.660	0.920	0.684
PRS07	1.154	1.155	0.789	0.841	1.174
CENG01	0.913	0.778	0.769	0.622	0.929
CENG02	0.921	0.921	0.954	0.921	1.013
CENG03	0.871	0.866	0.751	0.660	0.725
CENG04	1.021	1.070	0.882	0.854	0.871
CENG05	1.061	0.939	0.708	0.832	0.753
CENG06	1.037	0.831	0.513	0.666	0.804
CENG07	0.839	0.890	0.843	0.844	0.904
CENG08	0.770	0.767	0.633	0.596	0.779
EFFV01	-0.814	-0.603	-0.413	-0.524	-0.546
EFFV02	-1.077	-0.769	-0.337	-0.625	-0.617
EFFV03	-0.911	-0.635	-0.287	-0.399	-0.567

	EFC01	EFC02	EFC03	EFC04	PRS01
EFC01	2.350				
EFC02	1.285	2.504			
EFC03	1.801	1.494	2.652		
EFC04	1.439	1.584	1.706	2.320	
PRS01	-0.395	-0.675	-0.602	-0.649	2.163
PRS02	-0.221	-0.527	-0.447	-0.489	1.109
PRS03	-0.316	-0.573	-0.384	-0.471	1.090
PRS04	-0.241	-0.694	-0.346	-0.473	1.023

PRS05	-0.286	-0.547	-0.430	-0.535	1.238
PRS06	-0.249	-0.500	-0.357	-0.460	1.065
PRS07	-0.219	-0.656	-0.363	-0.467	1.017
CENG01	-0.035	-0.263	-0.215	-0.191	1.019
CENG02	-0.183	-0.524	-0.267	-0.338	1.027
CENG03	-0.088	-0.475	-0.359	-0.333	0.851
CENG04	-0.074	-0.500	-0.361	-0.384	0.953
CENG05	-0.245	-0.591	-0.345	-0.413	0.944
CENG06	-0.319	-0.470	-0.488	-0.600	0.940
CENG07	-0.062	-0.453	-0.245	-0.297	0.874
CENG08	-0.016	-0.355	-0.174	-0.193	0.748
EFFV01	0.541	0.739	0.599	0.664	-1.040
EFFV02	0.603	0.955	0.789	0.906	-1.036
EFFV03	0.547	0.806	0.758	0.827	-0.884

	PRS02	PRS03	PRS04	PRS05	PRS06
PRS02	1.666				
PRS03	1.033	1.832			
PRS04	0.853	0.962	2.013		
PRS05	1.010	1.218	0.829	1.885	
PRS06	0.906	1.102	0.809	1.188	1.646
PRS07	0.986	0.917	1.279	0.817	0.816
CENG01	0.836	0.882	0.837	0.725	0.621
CENG02	0.818	0.841	1.022	0.931	0.808
CENG03	0.816	0.742	1.013	0.728	0.722
CENG04	0.982	0.953	1.205	0.925	0.846
CENG05	0.815	0.892	0.848	0.922	0.752
CENG06	0.889	0.762	0.766	0.891	0.923
CENG07	0.790	0.822	0.997	0.777	0.716
CENG08	0.936	0.818	0.891	0.763	0.737
EFFV01	-0.663	-0.654	-0.541	-0.875	-0.764
EFFV02	-0.781	-0.714	-0.641	-0.849	-0.742
EFFV03	-0.794	-0.650	-0.519	-0.745	-0.659

	PRS07	CENG01	CENG02	CENG03	CENG04
PRS07	1.906				
CENG01	0.820	1.882			
CENG02	0.823	0.891	2.294		
CENG03	0.764	0.679	0.921	2.058	
CENG04	0.997	0.808	0.920	1.079	1.762
CENG05	0.801	0.751	0.905	0.722	0.898
CENG06	0.928	0.569	0.691	0.625	0.761
CENG07	0.982	0.806	0.912	0.778	0.987
CENG08	0.966	0.618	0.804	0.731	0.848
EFFV01	-0.619	-0.425	-0.650	-0.531	-0.495
EFFV02	-0.746	-0.439	-0.480	-0.496	-0.668
EFFV03	-0.771	-0.380	-0.361	-0.432	-0.614

	CENG05	CENG06	CENG07	CENG08	EFFV01

CENG05	1.967				
CENG06	0.672	1.895			
CENG07	0.983	0.663	1.875		
CENG08	0.776	0.632	0.804	1.920	
EFFV01	-0.589	-0.686	-0.402	-0.484	2.169
EFFV02	-0.753	-0.741	-0.521	-0.640	1.389
EFFV03	-0.574	-0.827	-0.475	-0.496	1.139

	EFFV02	EFFV03
EFFV02	2.502	
EFFV03	1.362	2.083

2.2 Covariance Matrix for the Female Sample

	II01	II02	II03	II04	II05
II01	2.774				
II02	1.659	2.520			
II03	1.108	0.958	1.992		
II04	1.456	1.104	1.117	2.713	
II05	1.330	1.211	0.904	0.825	2.224
EFC01	-0.582	-0.299	-0.361	-0.335	-0.342
EFC02	-1.446	-1.170	-0.985	-1.137	-0.886
EFC03	-0.925	-0.630	-0.550	-0.454	-0.674
EFC04	-1.310	-1.017	-0.911	-0.843	-0.933
PRS01	1.282	1.319	0.653	0.927	1.030
PRS02	1.107	0.952	0.688	0.835	0.876
PRS03	0.909	0.596	0.549	0.581	0.583
PRS04	1.042	0.987	0.879	0.825	1.071
PRS05	1.125	0.889	0.738	1.023	0.565
PRS06	1.006	0.863	0.668	0.952	0.611
PRS07	1.164	1.144	0.622	0.747	1.121
CENG01	0.836	0.653	0.609	0.520	0.804
CENG02	1.137	1.015	1.010	0.844	1.033
CENG03	0.771	0.683	0.593	0.340	0.693
CENG04	1.010	0.920	0.806	0.739	0.878
CENG05	0.770	0.671	0.388	0.600	0.515
CENG06	1.031	0.810	0.426	0.761	0.670
CENG07	0.605	0.576	0.667	0.771	0.784
CENG08	0.602	0.560	0.555	0.414	0.479
EFFV01	-0.865	-0.745	-0.465	-0.555	-0.549
EFFV02	-1.069	-0.915	-0.454	-0.796	-0.595
EFFV03	-0.929	-0.721	-0.424	-0.558	-0.684

	EFC01	EFC02	EFC03	EFC04	PRS01
EFC01	2.030				
EFC02	1.113	2.297			

EFC03	1.497	1.344	2.359		
EFC04	1.271	1.739	1.612	2.205	
PRS01	-0.562	-0.937	-0.811	-1.016	2.194
PRS02	-0.284	-0.828	-0.597	-0.826	1.086
PRS03	-0.497	-0.635	-0.458	-0.678	0.917
PRS04	-0.293	-0.798	-0.372	-0.623	0.967
PRS05	-0.486	-0.845	-0.600	-0.869	1.143
PRS06	-0.416	-0.723	-0.487	-0.644	1.030
PRS07	-0.303	-0.816	-0.455	-0.718	0.998
CENG01	-0.313	-0.446	-0.445	-0.557	0.942
CENG02	-0.450	-0.808	-0.593	-0.754	1.205
CENG03	-0.134	-0.602	-0.522	-0.530	0.708
CENG04	-0.203	-0.656	-0.601	-0.747	0.907
CENG05	-0.348	-0.678	-0.376	-0.559	0.708
CENG06	-0.334	-0.695	-0.519	-0.773	0.864
CENG07	-0.315	-0.547	-0.410	-0.500	0.841
CENG08	-0.053	-0.407	-0.162	-0.388	0.471
EFFV01	0.629	0.763	0.717	0.840	-1.250
EFFV02	0.518	0.983	0.773	0.931	-1.052
EFFV03	0.348	0.731	0.619	0.815	-1.008

	PRS02	PRS03	PRS04	PRS05	PRS06
PRS02	1.540				
PRS03	0.947	1.607			
PRS04	0.892	0.783	1.962		
PRS05	1.049	1.071	0.669	1.888	
PRS06	0.898	0.965	0.789	1.213	1.549
PRS07	0.941	0.772	1.271	0.813	0.730
CENG01	0.713	0.790	0.764	0.681	0.633
CENG02	0.917	0.804	1.072	1.002	1.017
CENG03	0.743	0.636	1.049	0.609	0.725
CENG04	0.992	0.763	1.174	0.850	0.788
CENG05	0.617	0.516	0.543	0.788	0.649
CENG06	0.864	0.543	0.721	0.894	0.756
CENG07	0.694	0.556	0.940	0.718	0.777
CENG08	0.853	0.682	0.750	0.801	0.688
EFFV01	-0.821	-0.799	-0.577	-1.124	-0.887
EFFV02	-0.890	-0.797	-0.587	-1.075	-0.880
EFFV03	-0.946	-0.759	-0.571	-0.944	-0.690

	PRS07	CENG01	CENG02	CENG03	CENG04
PRS07	1.861				
CENG01	0.730	1.870			
CENG02	0.869	0.832	2.344		
CENG03	0.742	0.697	0.959	2.005	
CENG04	0.911	0.687	0.983	1.124	1.861
CENG05	0.635	0.538	0.762	0.617	0.625
CENG06	0.759	0.470	0.830	0.524	0.747
CENG07	0.978	0.668	1.018	0.695	0.827

CENG08	0.673	0.359	0.743	0.658	0.837
EFFV01	-0.690	-0.499	-0.947	-0.493	-0.517
EFFV02	-0.807	-0.490	-0.588	-0.480	-0.811
EFFV03	-0.784	-0.422	-0.703	-0.515	-0.698

	CENG05	CENG06	CENG07	CENG08	EFFV01
CENG05	1.789				
CENG06	0.527	1.839			
CENG07	0.893	0.611	1.883		
CENG08	0.600	0.427	0.730	1.821	
EFFV01	-0.594	-0.731	-0.547	-0.497	2.101
EFFV02	-0.673	-0.863	-0.565	-0.574	1.336
EFFV03	-0.468	-0.821	-0.501	-0.458	1.106

	EFFV02	EFFV03
EFFV02	2.326	
EFFV03	1.159	1.622

2.3 Covariance Matrix for the Male Sample

	II01	II02	II03	II04	II05
II01	3.096				
II02	1.967	3.004			
II03	1.614	1.614	2.340		
II04	1.721	1.755	1.541	2.923	
II05	1.490	1.493	1.323	1.216	2.634
EFC01	-0.431	-0.336	-0.049	-0.153	0.024
EFC02	-1.113	-1.034	-0.635	-0.899	-0.666
EFC03	-0.721	-0.623	-0.168	-0.242	-0.197
EFC04	-0.848	-0.671	-0.262	-0.313	-0.330
PRS01	1.393	1.187	1.013	1.056	0.972
PRS02	1.113	0.935	0.847	0.710	0.812
PRS03	1.292	1.161	0.974	0.983	0.885
PRS04	1.217	1.314	1.021	0.987	1.121
PRS05	1.221	0.964	0.887	0.802	0.913
PRS06	1.023	0.829	0.637	0.878	0.740
PRS07	1.145	1.164	0.908	0.902	1.211
CENG01	0.966	0.890	0.851	0.672	1.028
CENG02	0.742	0.863	0.856	0.942	0.995
CENG03	0.957	1.006	0.896	0.932	0.748
CENG04	1.033	1.182	0.956	0.951	0.865
CENG05	1.292	1.144	0.973	1.022	0.939
CENG06	1.050	0.828	0.640	0.629	0.906
CENG07	1.031	1.139	0.995	0.915	1.003
CENG08	0.901	0.930	0.697	0.742	1.015
EFFV01	-0.781	-0.477	-0.414	-0.529	-0.543
EFFV02	-1.096	-0.634	-0.325	-0.547	-0.633

EFFV03	-0.913	-0.540	-0.282	-0.349	-0.473

	EFC01	EFC02	EFC03	EFC04	PRS01
EFC01	2.599				
EFC02	1.415	2.661			
EFC03	2.036	1.601	2.864		
EFC04	1.574	1.453	1.757	2.381	
PRS01	-0.261	-0.473	-0.453	-0.394	2.120
PRS02	-0.168	-0.281	-0.313	-0.204	1.142
PRS03	-0.172	-0.526	-0.327	-0.312	1.228
PRS04	-0.201	-0.621	-0.337	-0.377	1.051
PRS05	-0.126	-0.309	-0.296	-0.276	1.311
PRS06	-0.116	-0.324	-0.260	-0.325	1.085
PRS07	-0.160	-0.537	-0.298	-0.287	1.030
CENG01	0.186	-0.130	-0.050	0.072	1.061
CENG02	0.028	-0.307	-0.031	-0.040	0.861
CENG03	-0.053	-0.374	-0.223	-0.174	0.975
CENG04	0.029	-0.373	-0.167	-0.099	0.999
CENG05	-0.164	-0.519	-0.313	-0.294	1.133
CENG06	-0.303	-0.279	-0.443	-0.429	1.028
CENG07	0.134	-0.383	-0.116	-0.145	0.911
CENG08	0.014	-0.313	-0.182	-0.041	0.972
EFFV01	0.470	0.712	0.490	0.502	-0.899
EFFV02	0.667	0.919	0.770	0.841	-1.061
EFFV03	0.699	0.846	0.828	0.785	-0.840

	PRS02	PRS03	PRS04	PRS05	PRS06
PRS02	1.753				
PRS03	1.102	2.009			
PRS04	0.830	1.098	2.037		
PRS05	0.978	1.331	0.951	1.876	
PRS06	0.913	1.207	0.816	1.161	1.715
PRS07	1.027	1.027	1.280	0.816	0.884
CENG01	0.944	0.950	0.878	0.755	0.604
CENG02	0.756	0.866	0.962	0.870	0.633
CENG03	0.867	0.831	0.989	0.824	0.723
CENG04	0.972	1.104	1.235	0.986	0.893
CENG05	0.966	1.189	1.093	1.027	0.837
CENG06	0.889	0.939	0.820	0.889	1.064
CENG07	0.866	1.035	1.047	0.828	0.672
CENG08	0.995	0.925	1.002	0.732	0.773
EFFV01	-0.523	-0.543	-0.525	-0.681	-0.672
EFFV02	-0.667	-0.653	-0.708	-0.675	-0.642
EFFV03	-0.641	-0.572	-0.511	-0.593	-0.647

	PRS07	CENG01	CENG02	CENG03	CENG04
PRS07	1.946				
CENG01	0.883	1.875			

CENG02	0.779	0.921	2.229		
CENG03	0.780	0.672	0.899	2.095	
CENG04	1.071	0.910	0.878	1.044	1.686
CENG05	0.936	0.922	1.024	0.802	1.112
CENG06	1.075	0.666	0.609	0.694	0.768
CENG07	0.984	0.922	0.839	0.844	1.118
CENG08	1.199	0.824	0.854	0.784	0.857
EFFV01	-0.573	-0.383	-0.435	-0.551	-0.473
EFFV02	-0.707	-0.425	-0.430	-0.496	-0.546
EFFV03	-0.782	-0.386	-0.138	-0.350	-0.538

	CENG05	CENG06	CENG07	CENG08	EFFV01
CENG05	2.107				
CENG06	0.778	1.909			
CENG07	1.059	0.704	1.877		
CENG08	0.916	0.792	0.867	1.995	
EFFV01	-0.579	-0.628	-0.286	-0.471	2.207
EFFV02	-0.806	-0.601	-0.483	-0.688	1.400
EFFV03	-0.648	-0.779	-0.450	-0.525	1.126

	EFFV02	EFFV03
EFFV02	2.585	
EFFV03	1.452	2.352

3 A Mplus Output for the Structural Equation Modeling (SEM)

A Mplus output for the structural Equation Modeling(SEM) are provide below. The variable names used in the analysis are as follows: II = interest; EFC = effort cost; PRS = persistence, CENG = cognitive engagement; EFFV = effort avoidance.

INPUT INSTRUCTIONS

```
TITLE:      intefceng
DATA:       FILE IS 1.intefceng_mplus.dat;

DEFINE: ceng147 = MEAN (ceng01 ceng04 ceng07);
    ceng258 = MEAN (ceng02 ceng05 ceng08);
    ceng36 = MEAN (ceng03 ceng06);
    prs147 = MEAN (prs01 prs04 prs07);
    prs25 = MEAN (prs02 prs05);
    prs36 = MEAN (prs03 prs06);

VARIABLE: NAMES ARE sid sgc sch grd cls nmb sex age
     ii01-ii05 efc01-efc04 prs01-prs07 ceng01-ceng08
     effv01-effv03;
     Missing = ALL (-99);
```

```
     Usevariables =  ii01-ii05 efc01-efc04
      effv01-effv03 ceng147 ceng258 ceng36 prs147 prs25 prs36;

 MODEL:
    ii BY ii01-ii05;
    efc BY efc01-efc04;
    prs BY prs147 prs25 prs36;
    ceng BY ceng147 ceng258 ceng36;
    effv BY effv01-effv03;

    efc ON ii;
    prs ON ii efc;
    ceng ON ii efc;
    effv ON ii efc;

 MODEL INDIRECT:
    prs IND ii;
    ceng IND ii;
    effv IND ii;

 OUTPUT:      SAMPSTAT STANDARDIZED;

 INPUT READING TERMINATED NORMALLY
intefceng
SUMMARY OF ANALYSIS
Number of groups                                 1
Number of observations                         546
Number of dependent variables                   18
Number of independent variables                  0
Number of continuous latent variables            5



Observed dependent variables
 Continuous
  II01       II02       II03       II04       II05       EFC01
  EFC02      EFC03      EFC04      EFFV01     EFFV02     EFFV03
  CENG147    CENG258    CENG36     PRS147     PRS25      PRS36

Continuous latent variables
  II         EFC        PRS        CENG       EFFV


Estimator                                      ML
Information matrix                             OBSERVED
Maximum number of iterations                   1000
Convergence criterion                          0.500D-04
Maximum number of steepest descent iterations  20
Maximum number of iterations for H1            2000
Convergence criterion for H1                   0.100D-03
```

Input data file(s)
  1.intefceng_mplus.dat

Input data format FREE

SUMMARY OF DATA

Number of missing data patterns 14

COVARIANCE COVERAGE OF DATA

Minimum covariance coverage value 0.100

PROPORTION OF DATA PRESENT

Covariance Coverage

	II01	II02	II03	II04	II05
II01	1.000				
II02	0.995	0.995			
II03	0.998	0.993	0.998		
II04	0.996	0.993	0.995	0.996	
II05	0.993	0.987	0.991	0.989	0.993
EFC01	0.996	0.991	0.995	0.993	0.989
EFC02	0.991	0.985	0.989	0.987	0.984
EFC03	0.993	0.987	0.991	0.989	0.985
EFC04	0.991	0.985	0.989	0.987	0.984
EFFV01	0.998	0.993	0.996	0.995	0.991
EFFV02	0.996	0.991	0.995	0.993	0.989
EFFV03	0.998	0.993	0.996	0.995	0.991
CENG147	1.000	0.995	0.998	0.996	0.993
CENG258	1.000	0.995	0.998	0.996	0.993
CENG36	1.000	0.995	0.998	0.996	0.993
PRS147	1.000	0.995	0.998	0.996	0.993
PRS25	0.998	0.993	0.996	0.995	0.993
PRS36	1.000	0.995	0.998	0.996	0.993

Covariance Coverage

	EFC01	EFC02	EFC03	EFC04	EFFV01
EFC01	0.996				
EFC02	0.987	0.991			
EFC03	0.989	0.984	0.993		
EFC04	0.987	0.982	0.984	0.991	
EFFV01	0.995	0.989	0.991	0.989	0.998
EFFV02	0.993	0.987	0.989	0.987	0.995
EFFV03	0.995	0.989	0.991	0.989	0.996
CENG147	0.996	0.991	0.993	0.991	0.998
CENG258	0.996	0.991	0.993	0.991	0.998

CENG36	0.996	0.991	0.993	0.991	0.998
PRS147	0.996	0.991	0.993	0.991	0.998
PRS25	0.995	0.989	0.991	0.989	0.996
PRS36	0.996	0.991	0.993	0.991	0.998

Covariance Coverage

	EFFV02	EFFV03	CENG147	CENG258	CENG36
EFFV02	0.996				
EFFV03	0.995	0.998			
CENG147	0.996	0.998	1.000		
CENG258	0.996	0.998	1.000	1.000	
CENG36	0.996	0.998	1.000	1.000	1.000
PRS147	0.996	0.998	1.000	1.000	1.000
PRS25	0.995	0.996	0.998	0.998	0.998
PRS36	0.996	0.998	1.000	1.000	1.000

Covariance Coverage

	PRS147	PRS25	PRS36
PRS147	1.000		
PRS25	0.998	0.998	
PRS36	1.000	0.998	1.000

SAMPLE STATISTICS

ESTIMATED SAMPLE STATISTICS

Means

	II01	II02	II03	II04	II05
1	4.183	3.568	3.379	3.128	4.378

Means

	EFC01	EFC02	EFC03	EFC04	EFFV01
1	4.114	3.749	3.830	3.783	3.877

Means

	EFFV02	EFFV03	CENG147	CENG258	CENG36
1	3.121	2.922	4.441	4.296	4.418

Means

	PRS147	PRS25	PRS36
1	4.346	4.594	4.320

Covariances

II01	II02	II03	II04	II05

II01	2.955				
II02	1.828	2.795			
II03	1.401	1.306	2.251		
II04	1.613	1.456	1.402	2.867	
II05	1.418	1.367	1.137	1.044	2.452
EFC01	-0.498	-0.320	-0.186	-0.232	-0.138
EFC02	-1.261	-1.102	-0.776	-1.001	-0.765
EFC03	-0.808	-0.633	-0.312	-0.321	-0.407
EFC04	-1.045	-0.832	-0.511	-0.522	-0.592
EFFV01	-0.814	-0.603	-0.413	-0.522	-0.544
EFFV02	-1.077	-0.769	-0.337	-0.628	-0.616
EFFV03	-0.911	-0.636	-0.287	-0.399	-0.564
CENG147	0.925	0.912	0.832	0.774	0.901
CENG258	0.917	0.874	0.765	0.783	0.846
CENG36	0.953	0.838	0.620	0.669	0.748
PRS147	1.219	1.188	0.887	0.934	1.091
PRS25	1.155	0.941	0.794	0.831	0.807
PRS36	1.070	0.875	0.725	0.864	0.718

Covariances

	EFC01	EFC02	EFC03	EFC04	EFFV01
EFC01	2.349				
EFC02	1.287	2.508			
EFC03	1.801	1.496	2.652		
EFC04	1.440	1.586	1.707	2.321	
EFFV01	0.543	0.740	0.601	0.666	2.168
EFFV02	0.601	0.955	0.787	0.905	1.388
EFFV03	0.546	0.808	0.758	0.828	1.140
CENG147	-0.057	-0.406	-0.274	-0.292	-0.441
CENG258	-0.148	-0.491	-0.261	-0.315	-0.574
CENG36	-0.203	-0.461	-0.417	-0.461	-0.605
PRS147	-0.280	-0.676	-0.437	-0.529	-0.736
PRS25	-0.249	-0.537	-0.436	-0.512	-0.779
PRS36	-0.281	-0.536	-0.367	-0.465	-0.709

Covariances

	EFFV02	EFFV03	CENG147	CENG258	CENG36
EFFV02	2.502				
EFFV03	1.364	2.083			
CENG147	-0.542	-0.490	1.191		
CENG258	-0.622	-0.477	0.847	1.240	
CENG36	-0.618	-0.620	0.746	0.729	1.312
PRS147	-0.807	-0.724	0.968	0.899	0.876
PRS25	-0.822	-0.775	0.840	0.864	0.830
PRS36	-0.729	-0.656	0.807	0.808	0.785

Covariances

	PRS147	PRS25	PRS36
PRS147	1.420		

PRS25	0.979	1.398	
PRS36	0.946	1.086	1.422

Correlations

	II01	II02	II03	II04	II05
II01	1.000				
II02	0.636	1.000			
II03	0.543	0.521	1.000		
II04	0.554	0.514	0.552	1.000	
II05	0.527	0.522	0.484	0.394	1.000
EFC01	-0.189	-0.125	-0.081	-0.089	-0.057
EFC02	-0.463	-0.416	-0.327	-0.373	-0.308
EFC03	-0.289	-0.232	-0.128	-0.116	-0.159
EFC04	-0.399	-0.327	-0.223	-0.202	-0.248
EFFV01	-0.322	-0.245	-0.187	-0.210	-0.236
EFFV02	-0.396	-0.291	-0.142	-0.234	-0.249
EFFV03	-0.367	-0.263	-0.133	-0.163	-0.250
CENG147	0.493	0.500	0.508	0.419	0.527
CENG258	0.479	0.469	0.458	0.415	0.485
CENG36	0.484	0.438	0.361	0.345	0.417
PRS147	0.595	0.596	0.496	0.463	0.585
PRS25	0.568	0.476	0.447	0.415	0.436
PRS36	0.522	0.439	0.405	0.428	0.385

Correlations

	EFC01	EFC02	EFC03	EFC04	EFFV01
EFC01	1.000				
EFC02	0.530	1.000			
EFC03	0.721	0.580	1.000		
EFC04	0.617	0.658	0.688	1.000	
EFFV01	0.240	0.317	0.251	0.297	1.000
EFFV02	0.248	0.381	0.305	0.376	0.596
EFFV03	0.247	0.353	0.323	0.376	0.537
CENG147	-0.034	-0.235	-0.154	-0.176	-0.275
CENG258	-0.087	-0.278	-0.144	-0.186	-0.350
CENG36	-0.116	-0.254	-0.224	-0.264	-0.359
PRS147	-0.153	-0.358	-0.225	-0.292	-0.419
PRS25	-0.138	-0.287	-0.226	-0.284	-0.447
PRS36	-0.153	-0.284	-0.189	-0.256	-0.404

Correlations

	EFFV02	EFFV03	CENG147	CENG258	CENG36
EFFV02	1.000				
EFFV03	0.597	1.000			
CENG147	-0.314	-0.311	1.000		
CENG258	-0.353	-0.297	0.697	1.000	
CENG36	-0.341	-0.375	0.597	0.572	1.000
PRS147	-0.428	-0.421	0.744	0.678	0.642

PRS25	-0.439	-0.454	0.651	0.656	0.612
PRS36	-0.386	-0.381	0.620	0.609	0.574

Correlations

	PRS147	PRS25	PRS36
PRS147	1.000		
PRS25	0.695	1.000	
PRS36	0.666	0.770	1.000

MAXIMUM LOG-LIKELIHOOD VALUE FOR THE UNRESTRICTED (H1) MODEL IS -14318.881

UNIVARIATE SAMPLE STATISTICS

UNIVARIATE HIGHER-ORDER MOMENT DESCRIPTIVE STATISTICS

Variable/ Sample Size	Mean/ Variance	Skewness/ Kurtosis	Minimum/ Maximum	% with Min/Max	Percentiles 20%/60%	40%/80%	Median
II01	4.183	-0.224	1.000	7.33%	2.000	4.000	4.000
546.000	2.955	-0.965	7.000	6.78%	5.000	6.000	
II02	3.567	0.047	1.000	14.92%	2.000	3.000	4.000
543.000	2.798	-0.896	7.000	3.50%	4.000	5.000	
II03	3.376	0.234	1.000	12.29%	2.000	3.000	3.000
545.000	2.249	-0.467	7.000	2.57%	4.000	5.000	
II04	3.129	0.395	1.000	23.71%	1.000	3.000	3.000
544.000	2.869	-0.701	7.000	3.49%	4.000	5.000	
II05	4.384	-0.267	1.000	5.17%	3.000	4.000	4.000
542.000	2.454	-0.446	7.000	9.41%	5.000	6.000	
EFC01	4.118	-0.100	1.000	4.96%	3.000	4.000	4.000
544.000	2.346	-0.597	7.000	6.07%	5.000	5.000	
EFC02	3.756	0.114	1.000	7.21%	2.000	3.000	4.000
541.000	2.506	-0.771	7.000	4.25%	4.000	5.000	
EFC03	3.823	0.050	1.000	8.12%	2.000	3.000	4.000
542.000	2.648	-0.785	7.000	5.54%	4.000	5.000	
EFC04	3.784	0.121	1.000	6.10%	2.000	3.000	4.000
541.000	2.328	-0.647	7.000	4.07%	4.000	5.000	
EFFV01	3.877	0.023	1.000	4.77%	2.000	3.000	4.000
545.000	2.170	-0.630	7.000	3.85%	4.000	5.000	
EFFV02	3.118	0.574	1.000	15.99%	2.000	2.000	3.000
544.000	2.505	-0.306	7.000	4.04%	3.000	4.000	
EFFV03	2.921	0.632	1.000	16.88%	2.000	2.000	3.000
545.000	2.084	-0.071	7.000	1.65%	3.000	4.000	
CENG147	4.441	-0.196	1.000	0.37%	3.667	4.000	4.333
546.000	1.191	0.020	7.000	1.28%	4.667	5.333	
CENG258	4.296	-0.203	1.000	1.28%	3.333	4.000	4.333
546.000	1.240	0.263	7.000	1.10%	4.667	5.333	
CENG36	4.418	-0.070	1.000	0.55%	3.500	4.000	4.500
546.000	1.312	0.085	7.000	2.56%	4.500	5.500	
PRS147	4.346	-0.149	1.000	0.73%	3.333	4.000	4.333
546.000	1.420	-0.131	7.000	1.83%	4.667	5.333	

PRS25	4.598	-0.247	1.000	0.73%	4.000	4.500	4.500
545.000	1.390	0.185	7.000	3.85%	5.000	5.500	
PRS36	4.320	-0.064	1.000	0.73%	3.500	4.000	4.000
546.000	1.422	-0.156	7.000	2.38%	4.500	5.500	

THE MODEL ESTIMATION TERMINATED NORMALLY

MODEL FIT INFORMATION

Number of Free Parameters 64

Loglikelihood

H0 Value -14568.567
H1 Value -14318.881

Information Criteria

Akaike (AIC) 29265.134
Bayesian (BIC) 29540.502
Sample-Size Adjusted BIC 29337.340
(n* = (n + 2) / 24)

Chi-Square Test of Model Fit

Value 499.372
Degrees of Freedom 125
P-Value 0.0000

RMSEA (Root Mean Square Error Of Approximation)

Estimate 0.074
90 Percent C.I. 0.067 0.081
Probability RMSEA <= .05 0.000

CFI/TLI

CFI 0.936
TLI 0.922

Chi-Square Test of Model Fit for the Baseline Model

Value 6009.358
Degrees of Freedom 153
P-Value 0.0000

SRMR (Standardized Root Mean Square Residual)

Value 0.056

MODEL RESULTS

	Estimate	S.E.	Est./S.E.	Two-Tailed P-Value
**II BY**				
II01	1.000	0.000	999.000	999.000
II02	0.922	0.048	19.204	0.000
II03	0.752	0.045	16.746	0.000
II04	0.813	0.050	16.159	0.000
II05	0.754	0.047	16.015	0.000
**EFC BY**				
EFC01	1.000	0.000	999.000	999.000
EFC02	1.004	0.061	16.453	0.000
EFC03	1.155	0.057	20.289	0.000
EFC04	1.090	0.058	18.667	0.000
**PRS BY**				
PRS147	1.000	0.000	999.000	999.000
PRS25	0.958	0.038	25.001	0.000
PRS36	0.917	0.040	22.844	0.000
**CENG BY**				
CENG147	1.000	0.000	999.000	999.000
CENG258	0.975	0.043	22.465	0.000
CENG36	0.906	0.048	19.061	0.000
**EFFV BY**				
EFFV01	1.000	0.000	999.000	999.000
EFFV02	1.157	0.071	16.356	0.000
EFFV03	0.999	0.065	15.385	0.000
**EFC ON**				
II	-0.355	0.042	-8.550	0.000
**PRS ON**				
II	0.597	0.037	15.961	0.000
EFC	-0.014	0.035	-0.403	0.687
**CENG ON**				
II	0.537	0.035	15.207	0.000
EFC	0.059	0.034	1.727	0.084
**EFFV ON**				
II	-0.239	0.041	-5.782	0.000
EFC	0.351	0.050	6.965	0.000
**CENG WITH**				
PRS	0.321	0.033	9.668	0.000
**EFFV WITH**				

PRS	-0.308	0.042	-7.373	0.000
CENG	-0.188	0.037	-5.128	0.000

Intercepts

II01	4.183	0.074	56.857	0.000
II02	3.568	0.072	49.811	0.000
II03	3.378	0.064	52.600	0.000
II04	3.129	0.073	43.130	0.000
II05	4.377	0.067	65.159	0.000
EFC01	4.114	0.066	62.627	0.000
EFC02	3.752	0.068	55.266	0.000
EFC03	3.830	0.070	54.848	0.000
EFC04	3.784	0.065	57.918	0.000
EFFV01	3.878	0.063	61.495	0.000
EFFV02	3.121	0.068	46.049	0.000
EFFV03	2.922	0.062	47.291	0.000
CENG147	4.441	0.047	95.104	0.000
CENG258	4.296	0.048	90.150	0.000
CENG36	4.418	0.049	90.120	0.000
PRS147	4.346	0.051	85.212	0.000
PRS25	4.595	0.051	90.908	0.000
PRS36	4.320	0.051	84.639	0.000

Variances

II	1.946	0.177	10.965	0.000

Residual Variances

II01	1.010	0.083	12.099	0.000
II02	1.141	0.086	13.294	0.000
II03	1.150	0.081	14.271	0.000
II04	1.580	0.108	14.652	0.000
II05	1.348	0.092	14.622	0.000
EFC01	0.970	0.077	12.640	0.000
EFC02	1.111	0.084	13.191	0.000
EFC03	0.810	0.077	10.547	0.000
EFC04	0.681	0.064	10.559	0.000
EFFV01	0.993	0.080	12.436	0.000
EFFV02	0.928	0.087	10.637	0.000
EFFV03	0.908	0.076	11.973	0.000
CENG147	0.345	0.029	11.987	0.000
CENG258	0.436	0.033	13.267	0.000
CENG36	0.618	0.042	14.720	0.000
PRS147	0.344	0.027	12.534	0.000
PRS25	0.405	0.032	12.591	0.000
PRS36	0.518	0.038	13.561	0.000
EFC	1.136	0.122	9.322	0.000
PRS	0.371	0.041	9.054	0.000
CENG	0.323	0.039	8.336	0.000
EFFV	0.779	0.092	8.482	0.000

STANDARDIZED MODEL RESULTS

STDYX Standardization

	Estimate	S.E.	Est./S.E.	Two-Tailed P-Value
II BY				
II01	0.811	0.019	43.114	0.000
II02	0.769	0.021	36.436	0.000
II03	0.699	0.025	27.530	0.000
II04	0.670	0.027	24.903	0.000
II05	0.671	0.027	24.988	0.000
EFC BY				
EFC01	0.767	0.022	34.271	0.000
EFC02	0.746	0.023	31.979	0.000
EFC03	0.833	0.019	44.534	0.000
EFC04	0.841	0.018	47.113	0.000
PRS BY				
PRS147	0.871	0.013	68.872	0.000
PRS25	0.842	0.015	55.026	0.000
PRS36	0.797	0.018	43.533	0.000
CENG BY				
CENG147	0.843	0.016	53.263	0.000
CENG258	0.805	0.018	44.879	0.000
CENG36	0.727	0.022	32.326	0.000
EFFV BY				
EFFV01	0.736	0.026	28.798	0.000
EFFV02	0.793	0.023	34.323	0.000
EFFV03	0.751	0.025	30.001	0.000
EFC ON				
II	-0.422	0.043	-9.782	0.000
PRS ON				
II	0.802	0.030	26.885	0.000
EFC	-0.016	0.040	-0.403	0.687
CENG ON				
II	0.815	0.032	25.506	0.000
EFC	0.075	0.043	1.739	0.082
EFFV ON				
II	-0.307	0.050	-6.184	0.000
EFC	0.380	0.048	7.928	0.000
CENG WITH				
PRS	0.928	0.035	26.824	0.000

EFFV WITH

PRS	-0.572	0.053	-10.874	0.000
CENG	-0.374	0.062	-6.030	0.000

Intercepts

II01	2.433	0.085	28.570	0.000
II02	2.135	0.078	27.516	0.000
II03	2.252	0.080	27.979	0.000
II04	1.848	0.070	26.215	0.000
II05	2.795	0.095	29.364	0.000
EFC01	2.683	0.092	29.149	0.000
EFC02	2.371	0.084	28.272	0.000
EFC03	2.351	0.083	28.265	0.000
EFC04	2.483	0.087	28.664	0.000
EFFV01	2.633	0.090	29.105	0.000
EFFV02	1.973	0.073	26.847	0.000
EFFV03	2.025	0.075	27.087	0.000
CENG147	4.070	0.130	31.215	0.000
CENG258	3.858	0.124	31.027	0.000
CENG36	3.857	0.124	31.025	0.000
PRS147	3.647	0.118	30.810	0.000
PRS25	3.892	0.126	31.001	0.000
PRS36	3.622	0.118	30.782	0.000

Variances

II	1.000	0.000	999.000	999.000

Residual Variances

II01	0.342	0.031	11.187	0.000
II02	0.408	0.032	12.581	0.000
II03	0.511	0.036	14.392	0.000
II04	0.551	0.036	15.300	0.000
II05	0.550	0.036	15.242	0.000
EFC01	0.412	0.034	12.025	0.000
EFC02	0.444	0.035	12.746	0.000
EFC03	0.305	0.031	9.790	0.000
EFC04	0.293	0.030	9.777	0.000
EFFV01	0.458	0.038	12.154	0.000
EFFV02	0.371	0.037	10.111	0.000
EFFV03	0.436	0.038	11.594	0.000
CENG147	0.289	0.027	10.845	0.000
CENG258	0.351	0.029	12.153	0.000
CENG36	0.471	0.033	14.393	0.000
PRS147	0.242	0.022	11.007	0.000
PRS25	0.291	0.026	11.281	0.000
PRS36	0.364	0.029	12.468	0.000
EFC	0.822	0.036	22.636	0.000
PRS	0.345	0.036	9.472	0.000
CENG	0.382	0.040	9.530	0.000
EFFV	0.663	0.042	15.769	0.000

STDY Standardization

	Estimate	S.E.	Est./S.E.	Two-Tailed P-Value
II BY				
II01	0.811	0.019	43.114	0.000
II02	0.769	0.021	36.436	0.000
II03	0.699	0.025	27.530	0.000
II04	0.670	0.027	24.903	0.000
II05	0.671	0.027	24.988	0.000
EFC BY				
EFC01	0.767	0.022	34.271	0.000
EFC02	0.746	0.023	31.979	0.000
EFC03	0.833	0.019	44.534	0.000
EFC04	0.841	0.018	47.113	0.000
PRS BY				
PRS147	0.871	0.013	68.872	0.000
PRS25	0.842	0.015	55.026	0.000
PRS36	0.797	0.018	43.533	0.000
CENG BY				
CENG147	0.843	0.016	53.263	0.000
CENG258	0.805	0.018	44.879	0.000
CENG36	0.727	0.022	32.326	0.000
EFFV BY				
EFFV01	0.736	0.026	28.798	0.000
EFFV02	0.793	0.023	34.323	0.000
EFFV03	0.751	0.025	30.001	0.000
EFC ON				
II	-0.422	0.043	-9.782	0.000
PRS ON				
II	0.802	0.030	26.885	0.000
EFC	-0.016	0.040	-0.403	0.687
CENG ON				
II	0.815	0.032	25.506	0.000
EFC	0.075	0.043	1.739	0.082
EFFV ON				
II	-0.307	0.050	-6.184	0.000
EFC	0.380	0.048	7.928	0.000
CENG WITH				
PRS	0.928	0.035	26.824	0.000
EFFV WITH				

PRS	-0.572	0.053	-10.874	0.000
CENG	-0.374	0.062	-6.030	0.000

Intercepts

II01	2.433	0.085	28.570	0.000
II02	2.135	0.078	27.516	0.000
II03	2.252	0.080	27.979	0.000
II04	1.848	0.070	26.215	0.000
II05	2.795	0.095	29.364	0.000
EFC01	2.683	0.092	29.149	0.000
EFC02	2.371	0.084	28.272	0.000
EFC03	2.351	0.083	28.265	0.000
EFC04	2.483	0.087	28.664	0.000
EFFV01	2.633	0.090	29.105	0.000
EFFV02	1.973	0.073	26.847	0.000
EFFV03	2.025	0.075	27.087	0.000
CENG147	4.070	0.130	31.215	0.000
CENG258	3.858	0.124	31.027	0.000
CENG36	3.857	0.124	31.025	0.000
PRS147	3.647	0.118	30.810	0.000
PRS25	3.892	0.126	31.001	0.000
PRS36	3.622	0.118	30.782	0.000

Variances

II	1.000	0.000	999.000	999.000

Residual Variances

II01	0.342	0.031	11.187	0.000
II02	0.408	0.032	12.581	0.000
II03	0.511	0.036	14.392	0.000
II04	0.551	0.036	15.300	0.000
II05	0.550	0.036	15.242	0.000
EFC01	0.412	0.034	12.025	0.000
EFC02	0.444	0.035	12.746	0.000
EFC03	0.305	0.031	9.790	0.000
EFC04	0.293	0.030	9.777	0.000
EFFV01	0.458	0.038	12.154	0.000
EFFV02	0.371	0.037	10.111	0.000
EFFV03	0.436	0.038	11.594	0.000
CENG147	0.289	0.027	10.845	0.000
CENG258	0.351	0.029	12.153	0.000
CENG36	0.471	0.033	14.393	0.000
PRS147	0.242	0.022	11.007	0.000
PRS25	0.291	0.026	11.281	0.000
PRS36	0.364	0.029	12.468	0.000
EFC	0.822	0.036	22.636	0.000
PRS	0.345	0.036	9.472	0.000
CENG	0.382	0.040	9.530	0.000
EFFV	0.663	0.042	15.769	0.000

STD Standardization

	Estimate	S.E.	Est./S.E.	Two-Tailed P-Value
II BY				
II01	1.395	0.064	21.929	0.000
II02	1.285	0.063	20.331	0.000
II03	1.049	0.059	17.749	0.000
II04	1.134	0.067	16.811	0.000
II05	1.051	0.062	16.825	0.000
EFC BY				
EFC01	1.176	0.059	19.871	0.000
EFC02	1.180	0.062	19.190	0.000
EFC03	1.358	0.061	22.415	0.000
EFC04	1.281	0.056	22.906	0.000
PRS BY				
PRS147	1.037	0.041	25.236	0.000
PRS25	0.994	0.042	23.673	0.000
PRS36	0.951	0.044	21.768	0.000
CENG BY				
CENG147	0.920	0.039	23.582	0.000
CENG258	0.897	0.041	22.056	0.000
CENG36	0.833	0.044	19.120	0.000
EFFV BY				
EFFV01	1.084	0.059	18.382	0.000
EFFV02	1.255	0.062	20.218	0.000
EFFV03	1.084	0.058	18.812	0.000
EFC ON				
II	-0.422	0.043	-9.782	0.000
PRS ON				
II	0.802	0.030	26.885	0.000
EFC	-0.016	0.040	-0.403	0.687
CENG ON				
II	0.815	0.032	25.506	0.000
EFC	0.075	0.043	1.739	0.082
EFFV ON				
II	-0.307	0.050	-6.184	0.000
EFC	0.380	0.048	7.928	0.000
CENG WITH				
PRS	0.928	0.035	26.824	0.000
EFFV WITH				
PRS	-0.572	0.053	-10.874	0.000

CENG	-0.374	0.062	-6.030	0.000

Intercepts

II01	4.183	0.074	56.857	0.000
II02	3.568	0.072	49.811	0.000
II03	3.378	0.064	52.600	0.000
II04	3.129	0.073	43.130	0.000
II05	4.377	0.067	65.159	0.000
EFC01	4.114	0.066	62.627	0.000
EFC02	3.752	0.068	55.266	0.000
EFC03	3.830	0.070	54.848	0.000
EFC04	3.784	0.065	57.918	0.000
EFFV01	3.878	0.063	61.495	0.000
EFFV02	3.121	0.068	46.049	0.000
EFFV03	2.922	0.062	47.291	0.000
CENG147	4.441	0.047	95.104	0.000
CENG258	4.296	0.048	90.150	0.000
CENG36	4.418	0.049	90.120	0.000
PRS147	4.346	0.051	85.212	0.000
PRS25	4.595	0.051	90.908	0.000
PRS36	4.320	0.051	84.639	0.000

Variances

II	1.000	0.000	999.000	999.000

Residual Variances

II01	1.010	0.083	12.099	0.000
II02	1.141	0.086	13.294	0.000
II03	1.150	0.081	14.271	0.000
II04	1.580	0.108	14.652	0.000
II05	1.348	0.092	14.622	0.000
EFC01	0.970	0.077	12.640	0.000
EFC02	1.111	0.084	13.191	0.000
EFC03	0.810	0.077	10.547	0.000
EFC04	0.681	0.064	10.559	0.000
EFFV01	0.993	0.080	12.436	0.000
EFFV02	0.928	0.087	10.637	0.000
EFFV03	0.908	0.076	11.973	0.000
CENG147	0.345	0.029	11.987	0.000
CENG258	0.436	0.033	13.267	0.000
CENG36	0.618	0.042	14.720	0.000
PRS147	0.344	0.027	12.534	0.000
PRS25	0.405	0.032	12.591	0.000
PRS36	0.518	0.038	13.561	0.000
EFC	0.822	0.036	22.636	0.000
PRS	0.345	0.036	9.472	0.000
CENG	0.382	0.040	9.530	0.000
EFFV	0.663	0.042	15.769	0.000

R-SQUARE

Observed Variable	Estimate	S.E.	Est./S.E.	Two-Tailed P-Value
II01	0.658	0.031	21.557	0.000
II02	0.592	0.032	18.218	0.000
II03	0.489	0.036	13.765	0.000
II04	0.449	0.036	12.452	0.000
II05	0.450	0.036	12.494	0.000
EFC01	0.588	0.034	17.136	0.000
EFC02	0.556	0.035	15.989	0.000
EFC03	0.695	0.031	22.267	0.000
EFC04	0.707	0.030	23.557	0.000
EFFV01	0.542	0.038	14.399	0.000
EFFV02	0.629	0.037	17.162	0.000
EFFV03	0.564	0.038	15.001	0.000
CENG147	0.711	0.027	26.631	0.000
CENG258	0.649	0.029	22.440	0.000
CENG36	0.529	0.033	16.163	0.000
PRS147	0.758	0.022	34.436	0.000
PRS25	0.709	0.026	27.513	0.000
PRS36	0.636	0.029	21.766	0.000

Latent Variable	Estimate	S.E.	Est./S.E.	Two-Tailed P-Value
EFC	0.178	0.036	4.891	0.000
PRS	0.655	0.036	17.989	0.000
CENG	0.618	0.040	15.436	0.000
EFFV	0.337	0.042	8.031	0.000

QUALITY OF NUMERICAL RESULTS

Condition Number for the Information Matrix 0.196E-02
(ratio of smallest to largest eigenvalue)

TOTAL, TOTAL INDIRECT, SPECIFIC INDIRECT, AND DIRECT EFFECTS

	Estimate	S.E.	Est./S.E.	Two-Tailed P-Value
**Effects from II to PRS**				
Total	0.602	0.035	17.287	0.000
Total indirect	0.005	0.013	0.405	0.685
Specific indirect				
PRS				
EFC				
II	0.005	0.013	0.405	0.685
Direct				

```
   PRS
   II              0.597      0.037     15.961      0.000

Effects from II to CENG

  Total              0.517      0.032     16.141      0.000
  Total indirect    -0.021      0.013     -1.649      0.099

  Specific indirect

   CENG
   EFC
   II             -0.021      0.013     -1.649      0.099

  Direct
   CENG
   II              0.537      0.035     15.207      0.000

Effects from II to EFFV

  Total             -0.364      0.041     -8.932      0.000
  Total indirect    -0.125      0.022     -5.651      0.000

  Specific indirect

   EFFV
   EFC
   II             -0.125      0.022     -5.651      0.000

  Direct
   EFFV
   II             -0.239      0.041     -5.782      0.000

STANDARDIZED TOTAL, TOTAL INDIRECT, SPECIFIC INDIRECT, AND DIRECT EFFECTS

STDYX Standardization

                                                    Two-Tailed
                    Estimate       S.E.  Est./S.E.    P-Value

Effects from II to PRS

  Total              0.809      0.023     35.839      0.000
  Total indirect     0.007      0.017      0.406      0.685

  Specific indirect

   PRS
   EFC
   II              0.007      0.017      0.406      0.685
```

```
 Direct
  PRS
  II               0.802      0.030     26.885      0.000


Effects from II to CENG

 Total             0.783      0.025     30.933      0.000
 Total indirect   -0.032      0.019     -1.655      0.098

 Specific indirect

  CENG
  EFC
  II              -0.032      0.019     -1.655      0.098

 Direct
  CENG
  II               0.815      0.032     25.506      0.000


Effects from II to EFFV

 Total            -0.468      0.043    -10.951      0.000
 Total indirect   -0.160      0.026     -6.168      0.000

 Specific indirect

  EFFV
  EFC
  II              -0.160      0.026     -6.168      0.000

 Direct
  EFFV
  II              -0.307      0.050     -6.184      0.000


STDY Standardization

                                                Two-Tailed
                    Estimate       S.E.  Est./S.E.    P-Value

Effects from II to PRS

 Total             0.809      0.023     35.839      0.000
 Total indirect    0.007      0.017      0.406      0.685

 Specific indirect

  PRS
  EFC
  II               0.007      0.017      0.406      0.685
```

Direct
PRS
II 0.802 0.030 26.885 0.000

Effects from II to CENG

Total	0.783	0.025	30.933	0.000
Total indirect	-0.032	0.019	-1.655	0.098

Specific indirect

CENG
EFC
II -0.032 0.019 -1.655 0.098

Direct
CENG
II 0.815 0.032 25.506 0.000

Effects from II to EFFV

Total	-0.468	0.043	-10.951	0.000
Total indirect	-0.160	0.026	-6.168	0.000

Specific indirect

EFFV
EFC
II -0.160 0.026 -6.168 0.000

Direct
EFFV
II -0.307 0.050 -6.184 0.000

STD Standardization

	Estimate	S.E.	Two-Tailed Est./S.E.	P-Value

Effects from II to PRS

Total	0.809	0.023	35.839	0.000
Total indirect	0.007	0.017	0.406	0.685

Specific indirect

PRS
EFC
II 0.007 0.017 0.406 0.685

Direct
PRS
II 0.802 0.030 26.885 0.000

Effects from II to CENG

Total	0.783	0.025	30.933	0.000
Total indirect	-0.032	0.019	-1.655	0.098

Specific indirect

CENG
EFC
II -0.032 0.019 -1.655 0.098

Direct
CENG
II 0.815 0.032 25.506 0.000

Effects from II to EFFV

Total	-0.468	0.043	-10.951	0.000
Total indirect	-0.160	0.026	-6.168	0.000

Specific indirect

EFFV
EFC
II -0.160 0.026 -6.168 0.000

Direct
EFFV
II -0.307 0.050 -6.184 0.000
